# Inhibiting metabotropic glutamate receptor 5 after stroke restores brain function and connectivity

**DOI:** 10.1093/brain/awad293

**Published:** 2023-09-01

**Authors:** Jakob Hakon, Miriana J Quattromani, Carin Sjölund, Daniela Talhada, Byungchan Kim, Slavianka Moyanova, Federica Mastroiacovo, Luisa Di Menna, Roger Olsson, Elisabet Englund, Ferdinando Nicoletti, Karsten Ruscher, Adam Q Bauer, Tadeusz Wieloch

**Affiliations:** Division of Neurosurgery, Department of Clinical Sciences, Laboratory for Experimental Brain Research, Lund University, Lund 221 84, Sweden; Division of Neurosurgery, Department of Clinical Sciences, Laboratory for Experimental Brain Research, Lund University, Lund 221 84, Sweden; Division of Neurosurgery, Department of Clinical Sciences, Laboratory for Experimental Brain Research, Lund University, Lund 221 84, Sweden; Division of Neurosurgery, Department of Clinical Sciences, Laboratory for Experimental Brain Research, Lund University, Lund 221 84, Sweden; Department of Radiology, Washington University, Saint Louis, MO 63110, USA; Department of Molecular Pathology, IRCCS Neuromed, 86077 Pozzilli, Italy; Department of Molecular Pathology, IRCCS Neuromed, 86077 Pozzilli, Italy; Department of Molecular Pathology, IRCCS Neuromed, 86077 Pozzilli, Italy; Department of Experimental Medical Sciences, Chemical Biology & Therapeutics, Lund University, Lund 221 84, Sweden; Division of Pathology, Department of Clinical Sciences, Lund University, Lund 221 84, Sweden; Department of Molecular Pathology, IRCCS Neuromed, 86077 Pozzilli, Italy; Department of Physiology and Pharmacology, University of Rome La Sapienza, 00185 Rome, Italy; Division of Neurosurgery, Department of Clinical Sciences, Laboratory for Experimental Brain Research, Lund University, Lund 221 84, Sweden; Department of Radiology, Washington University, Saint Louis, MO 63110, USA; Division of Neurosurgery, Department of Clinical Sciences, Laboratory for Experimental Brain Research, Lund University, Lund 221 84, Sweden

**Keywords:** resting-state functional connectivity, stroke recovery, plasticity, pharmacological therapy, long term depression

## Abstract

Stroke results in local neural disconnection and brain-wide neuronal network dysfunction leading to neurological deficits. Beyond the hyper-acute phase of ischaemic stroke, there is no clinically-approved pharmacological treatment that alleviates sensorimotor impairments. Functional recovery after stroke involves the formation of new or alternative neuronal circuits including existing neural connections. The type-5 metabotropic glutamate receptor (mGluR5) has been shown to modulate brain plasticity and function and is a therapeutic target in neurological diseases outside of stroke. We investigated whether mGluR5 influences functional recovery and network reorganization rodent models of focal ischaemia.

Using multiple behavioural tests, we observed that treatment with negative allosteric modulators (NAMs) of mGluR5 (MTEP, fenobam and AFQ056) for 12 days, starting 2 or 10 days after stroke, restored lost sensorimotor functions, without diminishing infarct size. Recovery was evident within hours after initiation of treatment and progressed over the subsequent 12 days. Recovery was prevented by activation of mGluR5 with the positive allosteric modulator VU0360172 and accelerated in mGluR5 knock-out mice compared with wild-type mice. After stroke, multisensory stimulation by enriched environments enhanced recovery, a result prevented by VU0360172, implying a role of mGluR5 in enriched environment-mediated recovery. Additionally, MTEP treatment in conjunction with enriched environment housing provided an additive recovery enhancement compared to either MTEP or enriched environment alone. Using optical intrinsic signal imaging, we observed brain-wide disruptions in resting-state functional connectivity after stroke that were prevented by mGluR5 inhibition in distinct areas of contralesional sensorimotor and bilateral visual cortices. The levels of mGluR5 protein in mice and in tissue samples of stroke patients were unchanged after stroke.

We conclude that neuronal circuitry subserving sensorimotor function after stroke is depressed by a mGluR5-dependent maladaptive plasticity mechanism that can be restored by mGluR5 inhibition. Post-acute stroke treatment with mGluR5 NAMs combined with rehabilitative training may represent a novel post-acute stroke therapy.

## Teaser

Hakon *et al.* show that negative allosteric modulators of metabotropic glutamate receptor 5 (mGluR5 NAMs) restore lost sensorimotor function and functional connectivity in rodent models of focal ischaemia. Combined with rehabilitative training, mGluR5 NAMs may represent a novel post-acute stroke therapy.

## Introduction

Ischaemic stroke afflicts more than 12 million individuals world-wide, leads to 6 million deaths yearly and is a major cause of acquired long-term adult disability.^1^ Most stroke survivors exhibit some degree of spontaneous recovery, but the majority of patients report chronic motor^2^ and somatosensory^3^ deficits. Rehabilitation increases patient independence and participation,^4^ and small clinical studies have demonstrated improved outcome following targeted interventional strategies.^5^ However, because most large randomized clinical trials (RCTs) have been neutral to date,^6,7^ there remains an urgent medical need for post-acute stroke rehabilitative therapies.

While brain dysfunction after stroke appears to be due to structural damage, widespread functional disruptions are topographically linked within functional networks that can span across brain hemispheres.^8–11^ Brain dysfunction in areas remote from the lesion has been termed ‘diaschisis’,^12^ and more specifically as ‘connectomal diaschisis’ within the context of widespread changes in functional brain organization.^10,11^ Because brain networks affected by connectomal diaschisis potentially provide the substrate for functional recovery after stroke,^13–15^ it is necessary to consider more than just the ischaemic territory and its surround when characterizing molecular and systems-level mechanisms of brain repair.^16^

Functional neuroimaging studies reveal that patterns of resting state functional connectivity (RSFC) within and across resting state networks are altered after stroke.^17,18^ For example, disruption of interhemispheric homotopic RSFC predicts poor motor and attentional recovery.^19,20^ During the repair phase after stroke, renormalization of RSFC occurs in tandem with recovery and can involve tissue remodelling in both hemispheres^21–23^ features consistently observed across species.^24^ These repair processes are experience-dependent^25^ and, in the experimental setting, can be facilitated by task-specific sensorimotor training or multisensory stimulation in an enriched environment (EE).^26^ Housing in EEs after stroke strongly promotes recovery of sensorimotor functions,^27,28^ in particular tactile/proprioceptive paw placement (PP) functions, that are associated with brain network remodelling^29^ and increased spine density.^30^

The metabotropic glutamate receptor 5 (mGluR5) belongs to the group I mGluRs and is widely expressed throughout the CNS.^31^ mGluR5 is typically located at post-synaptic sites on glutamatergic pyramidal neurons as well as on GABAergic neurons and glial cells. Under physiological conditions, mGluR5 modulates synaptic transmission and might regulate both Hebbian and homeostatic plasticity.^32–34^ In pathological conditions, mGluR5 may contribute to maladaptive brain plasticity and persistent neurological dysfunctions^35^ as observed in Parkinson’s disease,^36^ fragile X syndrome (FXS)^37^ and Alzheimer’s disease.^38^ Negative allosteric modulators (NAMs) of mGluR5 have been in clinical trials as a potential therapeutic.^35^

In the context of stroke, we hypothesized that mGluR5 contributes to connectomal diaschisis and hampers recovery in the post-acute phase after injury. We show for the first time in rodent models of stroke that inhibiting the mGluR5 with NAMs in the post-acute phase after stroke accelerates functional brain organization and recovery of sensorimotor functions without diminishing brain damage. Applied as a standalone or adjunct treatment, mGluR5 NAMs present a novel strategy for enhancing recovery in stroke patients.

## Materials and methods

### Experimental design

**Figure 1:**
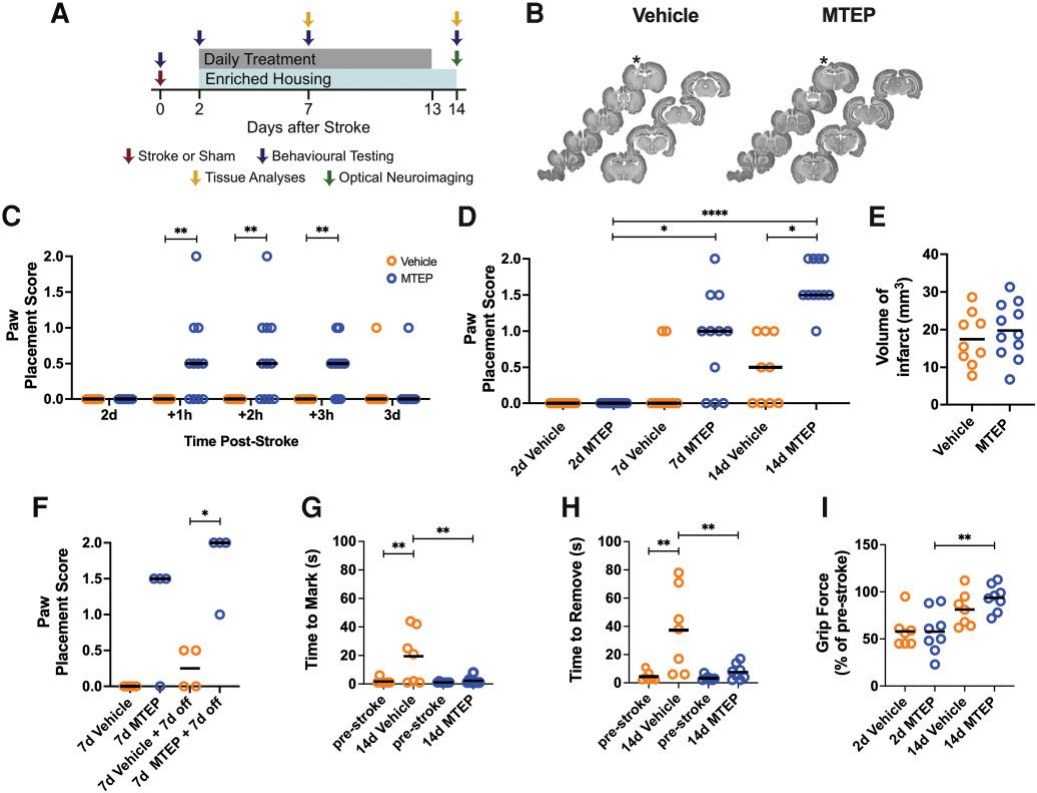
**Inhibition of mGluR5 improves lost sensorimotor function after photothrombotic stroke in rats.** (**A**) The study design. (**B**) Serial coronal NeuN stained sections of representative brains from Vehicle- (*left*) and MTEP- (*right*) treated rats subjected to photothrombotic (PT)-stroke. Cortical infarct is indicated by an asterisk. (**C**) Paw placement score at 2 days after PT-stroke and 1, 2 and 3 h and 3 days after the first injection (Vehicle, *n* = 9; MTEP, *n* = 11) [Kruskal–Wallis test with a *post hoc* Dunn’s multiple comparison (MC) test; **P < 0.01; bar denotes median]. (**D**) Paw placement at 2, 7 and 14 days after PT-stroke (Kruskal–Wallis test with a *post hoc* Dunn’s MC test; *P < 0.01, **P < 0.01, ***P < 0.001; bar denotes median). (**E**) Mean volume of infarct (mm^3^) assessed 14 days after stroke in Vehicle- (*n* = 9) or MTEP- (5 mg/kg, i.p., *n* = 11) treated rats (unpaired two-tailed *t*-test; bar denotes mean). (**F**) Paw placement in MTEP-treated animals 7 days and after an additional 7 days without (off) treatment compared to Vehicle (Kruskal–Wallis test with a *post hoc* Dunn’s MC test; *P < 0.05; bar denotes median). (**G** and **H**) Adhesive removal test. (**G**) Mean time (s) to mark and (**H**) mean time (s) to remove an adhesive on the left forepaw of Vehicle- (*n* = 7) or MTEP- (*n* = 8) treated rats; one-way ANOVA with a *post hoc* Sidak’s MC test; *P < 0.05, **P < 0.01; bar denotes mean. (**I**) Grip test. Mean grip force of right paw in % of pre-stroke force levels (Vehicle, *n* = 7; MTEP, *n* = 8); one-way ANOVA with a *post hoc* Sidak’s MC test; **P < 0.01; bar denotes mean. d = days.

In line with core recommendations for interventional studies in stroke recovery,^39^ our experiments incorporated several species of both genders, several stroke models, several behavioural tests and were performed in more than one laboratory. The design is depicted in Fig. 1A.

### Materials

MTEP (3-[(2-methyl-1,3-thiazol-4-yl)ethynyl]pyridine hydrochloride) was obtained from Beijing Honghui Meditech Co, Ltd. or Biotechne; VU360172 (*N*-cyclobutyl-6-[2-(3-fluorophenyl)ethynyl]-3-pyridinecarboxamide hydrochloride) and fenobam (*N*-(3-chlorophenyl)-*N*′-(4,5-dihydro-1-methyl-4-oxo-1H-imidazole-2-yl)urea from Beijing Honghui Meditech Co., Ltd. and AFQ056 (1H-indole-1-carboxylic acid, octahydro-4-hydroxy-4-(2-(3-methylphenyl)ethynyl) ethyl ester, (3aR,4S,7aR), from Sv Chembiotech. We confirmed the chemical structures of the compounds using H^1^-NMR; purity was >97%. The mGluR1 antagonist JNJ16259685 was from Biotechne; hydroxypropylmethylcellulose (HPMC) from Sigma and ^3^H-myoinositol from Perkin-Elmer.

### Animals

Our experiments included 193 male and 10 female C57BL/6 mice (Charles River), 69 male Sprague Dawley rats (Charles River) and 17 male Wistar rats (Charles River). Heterozygous B6; 129-Grm5^tm1Rod^/J mice were obtained from Jackson Laboratories (Stock number 003558). mGluR5^−/−^ and wild-type littermates were generated by heterozygous breeding. Animals were housed under a 12-h reversed light/dark cycle with free access to food and water. Behavioural analysis and image acquisition were performed during the awake periods. The photothrombotic (PT)-stroke studies were approved by the Malmö-Lund review committee (ethical permit number M 50-15 and M 25-12) and performed according to the ARRIVE guidelines.^40^ Every effort was made to reduce the number of animals used in the experiments according to the 3R (reduce, refine, replace) recommendation by the Swedish Department of Agriculture (www.djurforsok.info) and Swedish Research Council (www.vr.se). Animals were randomized prior to beginning experiments, and investigators were blinded to treatment and group assignment. Group and effect sizes and power were determined based on published and pilot data (Supplementary material). In the endothelin-1 (ET-1) model of middle cerebral artery occlusion (MCAO), all procedures were performed according to the guidelines of the Italian Ministry of Health (law 116/92) and EU Directive 86/609/EEC (authorization number, 1069/2015-PR). Human brain tissue use was approved by the Lund Ethical Review Board (Dnr 2011/80).

### Treatments

MTEP dissolved in vehicle (0.03% Tween 80 in saline) was injected intraperitoneally [5 mg/kg (4 ml/kg) unless otherwise stated]. The MTEP dose was chosen based on the reported 95% receptor occupancy in mice^41^ and 75–95% receptor occupancy in rats.^42^ This dose caused limited behavioural bias effects seen at higher doses.^42^ AFQ056, fenobam and VU0360172 were suspended in 0.5% hydroxy methyl propyl cellulose (HMPC) and sonicated for 3 min to a obtain a microsuspension. The pH was adjusted to pH 7.4 with NaOH. The doses were obtained from the literature and selected for their maximal pharmacological effect: AFQ056, 30 mg/kg p.o.^43^; VU0360172, 30 mg/kg p.o.^44,45^; fenobam, 30 mg/kg p.o.^36^; and the volume was 5 ml/kg.

To obtain groups with a similar functional deficit prior to treatment, selective sorting was performed on Day 2 after experimental stroke. Only animals exhibiting severe deficits in the PP test (score = 0; see ‘Behavioural tests’ section) were included. Approximately 13% of mice and rats were excluded by selective sorting. No animals were further excluded once assigned to a group.

### Enriched environment

EEs consisted of large cages containing different objects for exploration (i.e. tubes, ladders and platforms) with at least six mice/cage.^46^ Cages were cleaned and rearranged twice a week.^47^ The EE was initiated 2 days after PT-stroke. Additional mice were housed in standard cages (two mice per cage).

### Stroke models

The procedures for induction of PT-stroke in mice^46^ and rats^27^ and the ET-1 MCAO model^48^ are described in the Supplementary material.

### Behavioural tests

All behavioural testing was performed by the same blinded individual. The PP test assesses deficits in touch and proprioception (subdomains of somatosensation) in rats and mice^49^ as per our previous studies.^27,46^ The stroke lesion includes left primary motor and secondary motor areas and forelimb and hindlimb somatosensory cortical areas similar to our previous studies. This location of the lesion induces a robust, sensitive and persistent loss of PP after 2 days that remains for at least 9 weeks post-stroke.^27,49,50^ PP was tested by hand-holding each animal in a horizontal position allowing free movement of all four paws without receiving visual guidance or whisker contact cues. The animals were placed on a platform and moved laterally towards the edge of a platform until contact was lost with the platform, providing proprioceptive sensory stimulation (Supplementary Fig. 1A–F). Tactile stimulation was subsequently provided by lightly contacting the limbs with the platform’s edge. The movement of the paws towards the table top were registered. PP test scores for each limb were as follows: Score 1: quick placement of the limb on the table (Supplementary Fig. 1A); Score 0: no attempt to place the paws on platform leaving the limbs, paws and digits extended (Supplementary Fig. 1B); Score 0.5: incomplete placing of the limbs or paws were supinated and moved inwards towards the edge of the platform (Supplementary Fig. 1C–F).

In our previous studies on EE and stroke recovery, we reported a robust recovery enhancing effect of EE on PP functions after stroke (Supplementary material, p. 16). Since exploratory experiments indicated a similar response by MTEP treatment on PP function, PP was selected as the primary behavioural outcome measure. Also, since it is recommended to employ several tests of various sensorimotor functional domains in interventional studies of stroke recovery,^39^ we also employed an additional five behavioural tests.

The adhesive removal test^51^ is a sensorimotor test that detects sensory neglect in rats. Adhesive tape (3 × 4 mm) was applied to the palm of each forepaw and the rat placed in a transparent cylinder. Rats were accommodated to testing 4–5 days before stroke. Testing comprised three trials, each lasting maximum of 120 s. Performance was scored from recorded videos. For each trial, the tactile response was assessed as the latency to first contact of the adhesive by the snout or the time to start shaking the paws (touch). The motor response was the time between the first contact and the removal of the adhesive.

The grid sensorimotor and coordination test was performed mice, and the grip force test, beam walk sensorimotor and balance test, and postural hang reflex (PHR) laterality test were performed in rats and are described in the Supplementary material.

### OIS imaging

All mice were imaged 14 days after PT-stroke or sham surgery following previous reports.^29^ Anaesthesia was induced with an intraperitoneal injection of ketamine-xylazine (86.9/13.4 mg/kg; Ketaminol, Intervet/Rompun, Bayer). Mice were placed on a heating pad maintained at 37°C (mTCII, Cell Microcontrols), and fixed in a stereotactic frame. A midline incision was performed to expose the skull, which was kept moist by mineral oil. The OIS imaging system includes four light emitting diodes (LEDs) (470 nm, 590 nm, 617 nm, 625 nm; Thorlabs) placed around an EMCCD camera (iXon 897 Ultra, Andor Technologies) approximately 20 cm above the mouse’s head. Crossed linear polarizers prevented specular reflection off the skull during imaging. The camera was set to acquire 128 × 128 pixel images collected at 120 Hz (30 Hz/LED) using a custom-written software (MATLAB, Mathworks).

### OIS imaging data preprocessing

The data processing and analyses have been described previously.^29^ A brain mask for each mouse was used to define which pixels to process further.^52^ All imaging data were affine-transformed into a common mouse brain atlas. Imaging sessions with >1% temporal variation in mean light level intensity were excluded. Absorption data were converted into changes in oxy- and deoxy-haemoglobin concentration.^52,53^ Haemodynamic time traces were filtered between 0.009 and 0.08 Hz, then down-sampled from 30 to 1 Hz.^52,54^ Prior to RSFC analysis, mean global variance was regressed from all brain pixels in sham mice. For mice in the stroke groups, the average signals within the infarct and non-infarcted tissue were regressed simultaneously.^52^

### Resting state functional connectivity and network analyses

Global RSFC networks were evaluated via zero-lag correlation for all pixel pairs within the shared brain mask for each mouse according to published protocols.^55–57^ Spatial principal components analysis (PCA) was performed on the group averaged, whole cortex correlation difference matrix between Vehicle- and MTEP-treated mice 14 days after PT to evaluate RSFC topographies associated with the largest group-wise differences.

For graph analyses network nodes were determined by anatomical assignments defined by the Paxinos atlas (20 regions of interest in each hemisphere, 40 regions of interest in total). For each mouse, all pixel time traces within a given anatomical assignment were averaged and correlated with the corresponding time traces from all other assignments, creating a 40 × 40 connectivity matrix. For the network topology measures, we were most interested in examining network reorganization in preserved (non-infarct) cortical regions overlapping with the largest RSFC changes observed between MTEP and Vehicle-treated PT groups as determined by spatial PCA. Regions overlapping with lesioned tissue were therefore excluded from graph analyses. Cortical regions corresponding to the largest group wise differences were defined by thresholding the first principal component (PC1) at the 30th percentile (for all positive and negative values). This procedure resulted in 25 total nodes evaluated.

Each connectivity matrix was considered as a weighted undirected network, described by the graph *G*=(*V*, *W*), where *V* is the number of nodes (V = 25) and *W* is the number of edges. The weight matrix, *w_ij_* is a *V* × *V* symmetric weight matrix containing Fisher-*Z* transformed correlation coefficients. Self-connections (*w_ii_*) and negative correlation values (anticorrelations) were set to 0. Local and global functional network topology was assessed through the weighted undirected clustering coefficient and the shortest path length using the brain connectivity toolbox^58^ and as described.^59^

Maps of global node degree were calculated as described previously^29^ by thresholding whole cortex correlation matrices at *z*(r) ≥ 0. For each pixel (node) the number of intra- or inter-hemispheric connections were determined by summing over pixels in the ipsi- or contralesional hemisphere relative to the candidate pixel producing weighted maps of intra- and inter-hemispheric node degree. Group-level average maps were quantified within the following brain regions defined by the Paxinos atlas: primary motor (M1), secondary motor (M2), somatosensory forelimb (SFL), somatosensory hindlimb (SHL), posterior M2 (M2p), posterior- parietal (PP), retrosplenial (RS) and visual (VIS) cortex.^29^

### Measurement of polyphosphoinositide hydrolysis

Polyphosphoinositide (PI) hydrolysis was assessed by measuring inositol phosphate (InsP) accumulation in mouse cortical slices prelabelled with a tritiated precursor as described.^60^ Mice were decapitated, and the cortical tissue dissected on ice and transferred on ice-cold Krebs–Henseleit buffer (118 mM NaCl, 4.7 mM KCl, 1.18 mM MgSO_4_, 1.18 mM KH_2_PO_4_, 24.8 mM NaHCO_3_, 1.2 mM CaCl_2_, 10 mM D-glucose) pregassed with 95% O_2_ and 5% CO_2_ to pH 7.4. Slices (350 × 350 μm) prepared using a McIlwain tissue chopper were randomly distributed into different tubes. Forty microlitres of gravity packed slices/tube were incubated for 60 min in 350 μl buffer containing 1 μCi of ^3^H-myo-inositol. Slices were incubated with LiCl (10 mM to block InsP degradation) and with the mGlu1 receptor antagonist JNJ16259685 (10 μM to block mGlu1 receptors). Thereafter, slices were incubated with Vehicle or the group I mGlu receptor agonist DHPG (200 μM). Incubations were stopped after 60 min by adding 900 μl methanol/chloroform (2:1). After further addition of 300 μl chloroform and 600 μl water, samples were centrifuged at low speed to facilitate phase separation, and the [^3^H]InsP present in the supernatant was separated by anion exchange chromatography. Samples were removed from their water phase, incubated with 0.5 N NaOH and allowed to dry at 50°C for 2 h. Protein concentration was assessed and radioactivity counted.

### Infarct volume

Infarct volume was determined as previously described^29^ (Supplementary material).

### Western blot analysis

For western blot analysis and immunohistochemistry, see the Supplementary material.

### Statistical analysis

Statistical analysis and figure preparation was performed in MATLAB (Mathworks), PRISM 9 (Graph Pad), G*Power (University of Dusseldorf) and Illustrator 2023 (Adobe). For all plots, individual data are denoted, and horizontal bars indicate mean values for parametric data sets and median values for non-parametric data. No statistical outliers were excluded. Standard statistical testing was applied for evaluating group-wise differences (Supplementary material), and data classifications and experiment-specific statistical tests are listed in Supplementary Table 4. A *P* < 0.05 was considered statistically significant, and for all figures, *P < 0.05, **P < 0.01 and ***P < 0.001. Calculations of sample sizes, effect sizes and power are presented in detail in the Supplementary material.

## Results

### Negative allosteric modulators of mGluR5 enhance recovery of sensorimotor function

The PT lesion involved most of the left primary sensorimotor cortex down to the corpus callosum and was positioned to cause a reproducible and robust deficit in PP ability^49^ (Fig. 1B). Before stroke, all animals performed with a maximal PP test score (PP score = 2). Two days after stroke, when infarct development subsided, only rodents with severe PP deficit (PP score = 0) of the right limbs were included (Supplementary Fig. 1B). The left paws were unaffected by stroke, with a PP score of 2 in all studies.

We first treated rats with Vehicle or MTEP starting 2 days after stroke. Moderate but significant recovery of PP function was evident 1 h after the first MTEP injection, which persisted for 2 h and then subsided by 24 h after the injection (Fig. 1C; *P* < 0.01). With daily MTEP treatment, recovery progressively improved; at 14 days post-stroke, 10 of 11 animals had a PP test score >1. Vehicle-treated animals remained severely impaired with PP test scores of 1 or less (Fig. 1D; *P* < 0.01). There was no difference in infarct size between the two groups (Fig. 1E; Vehicle: 17.5 ± 6.9 mm^3^; MTEP: 19.8 ± 7.4 mm^3^). The benefit of treatment was maintained 7 days after termination of MTEP treatment (Fig. 1F; *P* < 0.01).

In the adhesive removal test, pre-stroke rats marked the adhesive within 1.7 ± 0.7 s (Vehicle) and 1.1 ± 0.1 s (MTEP) and removed it within 2.7 ± 1.3 s (Vehicle) and 2.1 ± 0.6 s (MTEP). At 14 days of recovery, the time to mark increased to 19 ± 7 s in the Vehicle-treated group, signifying tactile extinction, while it was significantly faster in the MTEP-treated group (2.4 ± 0.9 s) (Fig. 1G; *P* < 0.05). The time to remove the adhesive was 37.3 ± 11.2 s in the Vehicle group and five times faster (7.63 ± 2 s) in the MTEP group (Fig. 1H). In the same experimental series, the grip force assessed at 2 days of recovery was reduced by 40% in both treatment groups. After 12 days of treatment, force recovered significantly to near pre-stroke values in the MTEP-treated but not in Vehicle-treated groups. Still, there was no significant difference between the treatment groups (Fig. 1I; *P* < 0.01).

**Figure 2:**
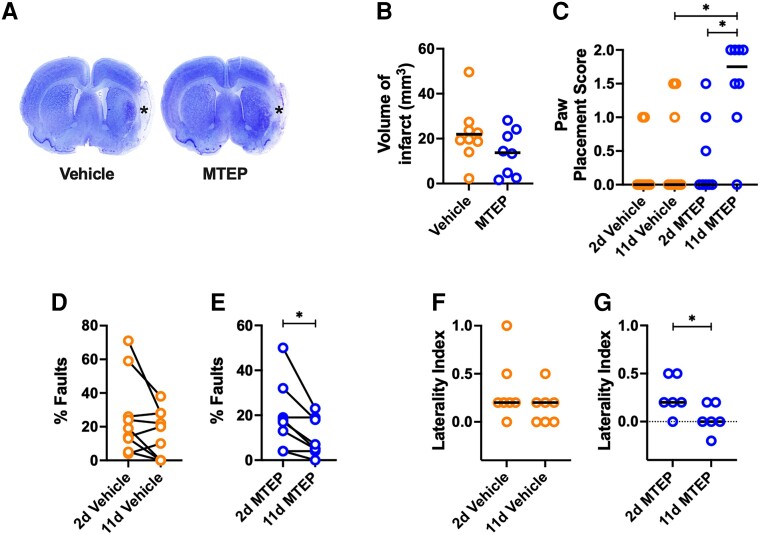
**Inhibition of mGluR5 enhances recovery of sensorimotor function in the endothelin-1 middle cerebral artery occlusion model of stroke in rats**. (**A**) Nissl-stained coronal sections of rat brains displaying brain infarcts (asterisk) caused by endothelin-1 (ET-1) injected onto the middle cerebral artery in Vehicle- or MTEP-treated rats. (**B**) Mean volume of infarct (mm^3^) (*t*-test; bar denotes mean) and (**C**) paw placement score at 11 days after stroke (Vehicle, *n* = 9; MTEP, *n* = 8; Kruskal–Wallis test with a *post hoc* Dunn’s multiple comparison test; *P < 0.05; bar denotes median). (**D** and **E**) Foot faults (% of total number of steps) on an elevated beam after daily (**D**) Vehicle- (*n* = 9) or (**E**) MTEP- (5 mg/kg, i.p., *n* = 8) treatment (paired *t*-test; *P < 0.05). The laterality index obtained from the postural hang reflex test after daily treatment with (**F**) Vehicle (*n* = 7) or (**G**) MTEP (5 mg/kg, i.p., *n* = 6) (Wilcoxon’s matched-paired signed test; *P < 0.05). d = days.

The effects of MTEP were also evaluated in the ET-1 model of MCAO, which causes larger lesions involving lateral aspects of cortical sensorimotor regions and sometimes subcortical tissue (Fig. 2A). There was no difference in infarct size between Vehicle- (24.3 ± 13.3 mm^3^) and MTEP-treated animals (18.2 ± 10 mm^3^) (Fig. 2B) following MCAO. The PP scores in the MTEP-treated animals improved significantly compared to the Vehicle-treated animals after ET-1 stroke, assessed at 11 days of treatment (Fig. 2C; *P* < 0.05). In the beam walk foot fault-test the MTEP-treated performed with fewer foot faults but not the Vehicle-treated group (Fig. 2D and E; *P* < 0.05). Also, in the PHR laterality test, an increase in laterality index was evident in the Vehicle-treated animals at 2 and 11 days post-stroke, while in the MTEP-treated group, laterality index significantly improved at 11 days compared with 2 days of recovery (Fig. 2F and G)

**Figure 3:**
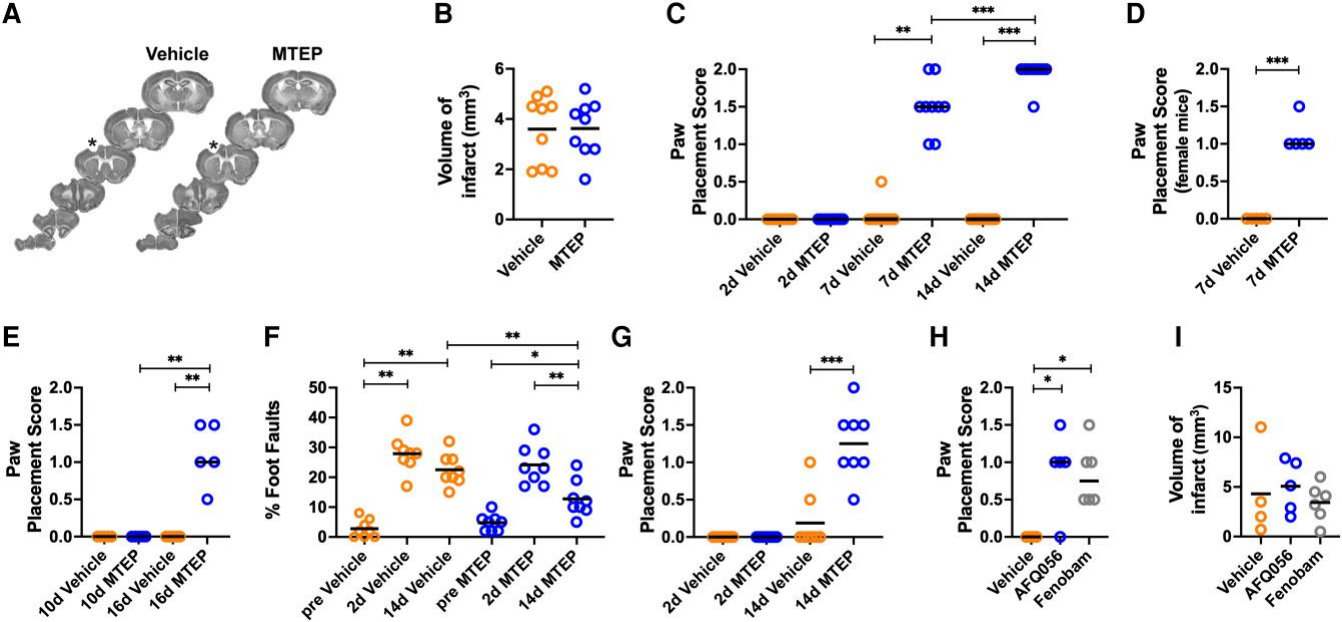
**Inhibition of mGluR5 improves lost sensorimotor function after photothrombotic stroke in mice.** (**A**) Serial coronal mouse brain slices from Vehicle- and MTEP-treated stroke groups at 14 days after photothrombotic (PT)-stroke. Asterisk indicates infarct. (**B**) Mean volume of infarct (mm^3^) (unpaired two-tailed *t*-test; bar denotes mean) and (**C**) paw placement score in Vehicle- (*n* = 9) or MTEP- (5 mg/kg, i.p., *n* = 9) treated mice at 7 and 14 days after stroke [Kruskal–Wallis test with a *post hoc* Dunn’s multiple comparison (MC) test; **P < 0.01, ***P < 0.001; bar denotes median]. (**D**) Paw placement score of female mice at 7 days after stroke treated with Vehicle (*n* = 5) or MTEP (5 mg/kg, i.p., *n* = 5) (Mann–Whitney test; ***P < 0.001; bar denotes median). (**E**) Paw placement score at 10 and 16 days after stroke in mice treated daily with Vehicle (*n* = 5) or MTEP (5 mg/kg, i.p., *n* = 5) starting on Day 10 after stroke. All mice had a score of 2 before stroke induction. (Kruskal–Wallis test with a *post hoc* Dunn’s MC test; ***P < 0.001; bar denotes median). (**F**) Foot fault test. Percentage of faults by Vehicle- (*n* = 8) or MTEP-treated mice (5 mg/kg, i.p. daily, *n* = 8) at 2 and 14 days after stroke (one-way ANOVA with a *post hoc* Sidak’s MC test; *P < 0.05, **P < 0.01, ***P < 0.001; bar denotes mean). (**G**) Paw placement score of animals in **F** (Kruskal–Wallis test with a *post hoc* Dunn’s MC test; ***P < 0.001; bar denotes median). (**H**) Paw placement score at 7 days after PT-stroke of mice treated daily with Vehicle (*n* = 4), AFQ056 (*n* = 5; 30 mg/kg, p.o., *n* = 5) or fenobam (30 mg/kg p.o.; *n* = 6; Kruskal–Wallis test with a *post hoc* Dunn’s MC test; *P < 0.05; bar denotes median). (**I**) Mean volume of infarct (mm^3^) of animals reported in **H**. No difference between groups was found (one-way ANOVA; bar denotes mean). d = days.

MTEP treatment also improved post-stroke sensorimotor functions in mice. Figure 3A displays the position of the infarct in the mouse brain following PT stroke. At 14 days after PT stroke, treatment with MTEP did not affect infarct size (Fig. 3B; Vehicle: 3.6 ± 0.5 mm^3^ and MTEP: 3.6 ± 0.4 mm^3^). Vehicle-treated mice displayed a marked PP deficit, while in MTEP-treated mice, PP function progressively improved to almost full recovery (Fig. 3C; *P* < 0.001). The recovery enhancing effect of MTEP at 7 days after stroke in female mice was of similar magnitude as that in males at this time of recovery (Fig. 3D; *P* < 0.001). To assess the therapeutic window of mGluR5 NAMs, MTEP treatment was delayed and initiated 10 days after stroke and continued for 6 days. PP significantly improved in the MTEP-treated group (Fig. 3E; *P* < 0.05).

In the grid test, the fraction of foot faults of the right sided paws increased from 2.8 ± 1% of the total number of steps before stroke to 28 ± 2% in the Vehicle group, and from 4.8 ± 1% to 24 ± 2% in the MTEP group at 2 days of recovery (Fig. 3F). After 12 days of treatment, the fraction of foot faults remained elevated at 22.5 ± 1.9% in the Vehicle-treated group, while in the MTEP-group, the foot faults decreased to 12.8 ± 2%, i.e. 56% less than in the Vehicle-treated group (Fig. 3F; *P* < 0.05). In the same experimental series, the MTEP-treated animals improved in the PP test (Fig. 3G; *P* < 0.001). Although multiple behavioural tests demonstrate improved recovery of sensorimotor functions following MTEP treatment, the PP test, our primary outcome measure, was used in subsequent studies due to its robustness and sensitivity.^27,29,46^

AFQ056 and fenobam are low molecular weight mGluR5 NAMs with chemical structures distinct from MTEP. The compounds cross the blood–brain barrier when administered orally and have been employed in clinical studies for brain disorders other than stroke.^31^ In mice, treatment with AFQ056 or fenobam, starting 2 days after PT-stroke, enhanced recovery of PP function (Fig. 3H; *P* < 0.05) without affecting infarct size (Fig. 3I).

**Figure 4:**
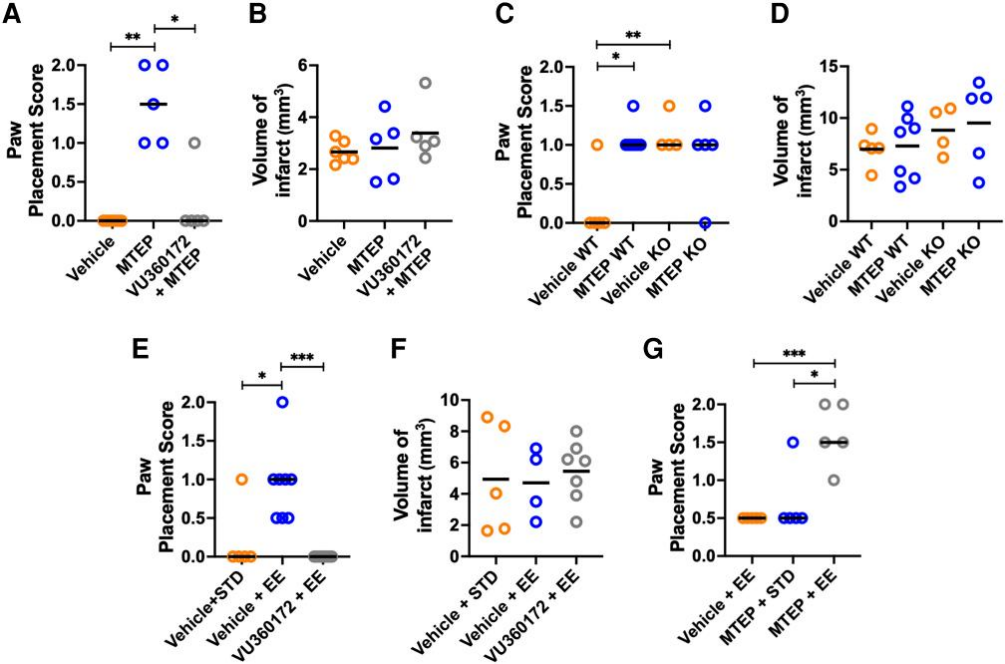
**mGluR5 is involved in multisensory assisted recovery of sensorimotor function after stroke in mice.** (**A**) Daily pretreatment with the mGluR5 positive allosteric modulator VU360172 (30 mg/kg p.o. daily, *n* = 5) 1 h prior to MTEP (5 mg/kg, i.p.) prevents the recovery enhancing effect of MTEP (*n* = 5) compared to Vehicle (*n* = 6) at 7 days post stroke. [Kruskal–Wallis test with a *post hoc* Dunn’s multiple comparison (MC) test; *P < 0.05, **P < 0.01; bar denotes median]. (**B**) Mean volume of infarct (mm^3^) of animals in **A**. No difference between groups was found (one-way ANOVA; bar denotes mean). (**C**) Genetic deletion of mGluR5 mimics treatment with MTEP. Wild-type (WT) mice were treated with Vehicle (*n* = 5) or MTEP (*n = 7*) and mGluR5 knock-out (KO) mice treated with Vehicle (*n* = 4) or MTEP (*n* = 5) from 2 to 7 days post-stroke (Kruskal–Wallis test with a *post hoc* Dunn’s MC test; *P < 0.05, **P < 0.01; bar denote median). (**D**) Mean volume of infarct (mm^3^) of animals in **C**. No difference between groups was found (one-way ANOVA; bar denotes mean). (**E**) The recovery enhancing effect of enriched environment (EE) at 7 days post-stroke (*n* = 8) is prevented by concomitant treatment with VU360172 (30 mg/kg p.o., *n* = 7) for 5 days (Kruskal–Wallis test with a *post hoc* Dunn’s MC test *P < 0.05, ***P < 0.001; bar denotes median). (**F**) Volume of infarct (mm^3^) of animals in **E**. No difference between groups was found (one-way ANOVA; bar denotes mean). (**G**) Paw placement score of mice subjected to photothrombotic (PT) stroke and treated daily with MTEP (1 mg/kg i.p., *n* = 5) and concomitantly housed in either standard cages (STD) or in an enriched environment (EE) (*n* = 5) from 2 to 7 days post-stroke display an additive recovery effect of the combination treatment (MTEP + EE, *n* = 5) (Kruskal–Wallis test with a *post hoc* Dunn’s MC test; *P < 0.05, **P < 0.01; bar denotes median).

To further validate the action of MTEP on the mGlu5 receptor, mice were treated with a positive allosteric modulator (PAM) of mGluR5 VU0360172^44,45^ daily starting 2 days after stroke and 1 h prior to MTEP treatment (Fig. 4A). In the group treated with MTEP alone, PP significantly improved at 7 days post-stroke (*P* < 0.01), while in the group treated with Vehicle alone, PP scores remained depressed. Pretreatment with VU0360172 almost completely prevented the recovery effect of MTEP (*P* < 0.05). There was no difference in infarct size among the groups (Fig. 4B).

We next asked whether deletion of the mGluR5 gene (*GRM5*) in mice would influence the dynamics of recovery (Fig. 4C). In Vehicle-treated wild-type mice, PP remained depressed after stroke, while compared to this group, both Vehicle-treated mGluR5 knock-out mice (*P* < 0.01) as well as MTEP-treated wild-type mice (*P* < 0.05) had higher PP scores. Infarct size did not differ across the groups (Fig. 4D).

We next examined whether mGluR5 is involved in EE-mediated recovery of PP function after stroke. In accordance with our earlier studies,^29,46^ housing mice in EE starting 2 days after stroke significantly improved PP functions compared with mice housed in standard cages (Fig. 4E; *P* < 0.05). Daily treatment with VU0360172 prevented the recovery enhancing effect of the EEs (*P* < 0.001), without affecting infarct size (Fig. 4F), implying the involvement of mGluR5 in the recovery enhancing action of EE exposure.

We next explored the effect of combining EE and MTEP treatment. Combining the EE with a submaximal dose of MTEP (1 mg/kg i.p. daily) provided an additive significant improvement in PP (Fig. 4G; *P* < 0.05). Hence, inhibition of mGluR5 acts in concert with the EE to promote recovery.

Taken together, mGluR5 hampers early post-stroke recovery processes. The loss of sensorimotor functions, particularly tactile/proprioceptive PP deficits, following experimental stroke are restored by inhibiting mGluR5 in the post-acute phase. Activation of mGluR5 prevents recovery stimulated by the EE, while blocking mGluR5 in conjunction with EE enhances the beneficial effects of multisensory stimulation on recovery. The lack of pharmacological effects of mGluR5 NAM treatment in uninjured animals implies that injury is required for mGluR5-dependent deficits. Because all groups treated with or without mGluR5 NAMs exhibited the same infarct size, the behavioural restorative effects of mGluR5 inhibition were not due to neuroprotection.

### MTEP-induced recovery of neurological functions is associated with increased intrahemispheric RSFC

**Figure 5:**
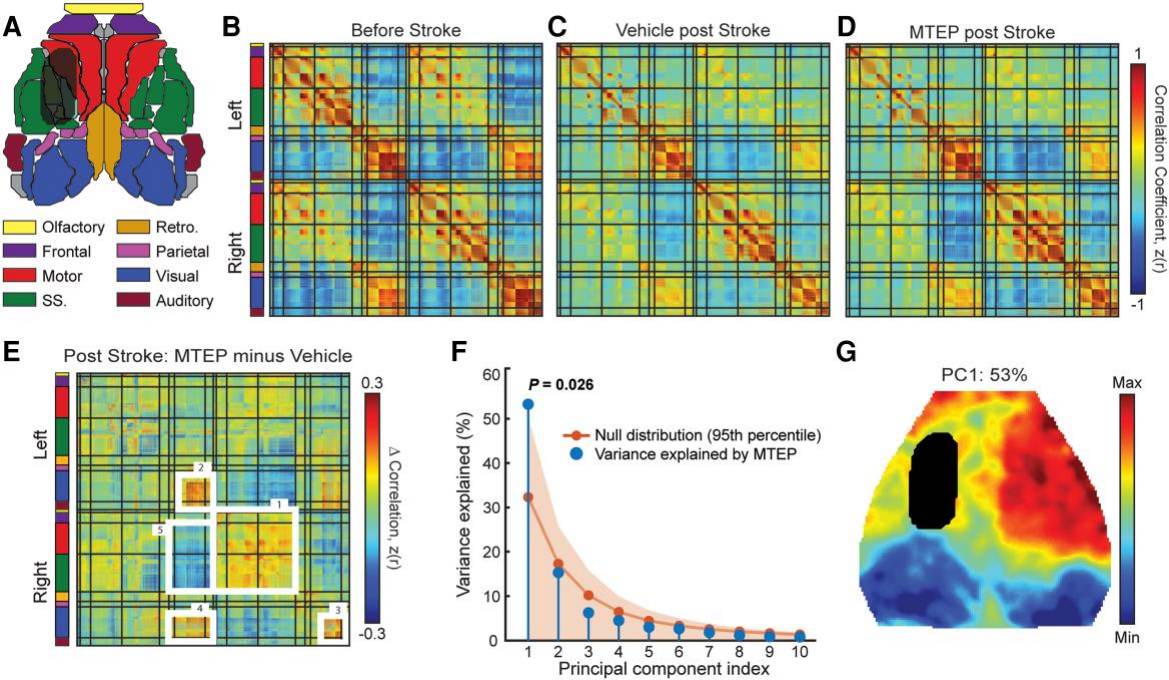
**Inhibition of mGluR5 improves functional brain organization following stroke.** (**A**) Field of view of optical intrinsic signal imaging system used for mapping functional connectivity in the mouse with and without stroke. Coloured parcels indicate assignments according to the Paxinos mouse brain atlas. The infarct is indicated in the left hemisphere. (**B**–**D**) Group-averaged, whole-cortex correlation matrices for (**B**) control mice (i.e. sham mice treated with Vehicle, *n* = 5) and 14 days after photothrombotic (PT)-stroke for (**C**) mice treated with Vehicle (*n* = 10) or (**D**) mice treated with MTEP (*n* = 10). Group level correlation matrices show all pairwise resting state functional connectivity (RSFC) within our field of view. Matrices are grouped by functional assignment and organized by hemisphere (left, ipsilesional; right contralesional). Difference correlation matrix (MTEP minus Vehicle) shows the group-averaged correlation differences between the MTEP- and Vehicle-treated groups. (**E**) After PT-stroke, MTEP-treated mice exhibit higher intrahemispheric RSFC within contralesional somatosensory, motor and surrounding cortices (box 1), ipsilesional (box 2) and contralesional (box 3) visual cortices, and stronger homotopic RSFC in visual regions (box 4). Further, larger anticorrelations between anterior-posterior brain regions were also more pronounced in MTEP-treated mice compared with Vehicle-treated mice (box 5). All matrices are reported as Fisher *z*-scores. (**F**) Eigenspectrum following spatial principal component analysis (PCA) of the group-level correlation difference matrix in **E**. Blue dots represent the variance explained by the first 10 PCs, plotted in descending order according to their contribution to the overall eigenspectrum. Extensive permutation resampling (3000 iterations) of all mice between groups was used to determine the amount of variance explained by the first eigenvalue in the null case. The orange dots represent the average variance explained by individual PCs across all 3000 iterations, while the shaded orange region represents the 95th percentile of the null distribution for each PC. In the true eigenspectrum (i.e. RSFC differences between MTEP PT and Vehicle PT mice), the variance explained by PC1 was in the 97.45th percentile (i.e. *P* = 0.0255) and was considered statistically significant. (**G**) Topography of PC1 reveals increased, positive, contralateral functional connectivity in motor and sensory regions, as well as increased anticorrelations between anterior-posterior brain regions.

Functional recovery after stroke is associated with restoration of RSFC within affected and distant brain regions,^21,24^ and EE exposure accelerates these processes.^29^ We used optical intrinsic signal imaging before and 14 days after PT in mice^29,61^ to determine whether MTEP treatment affected systems-level brain organization after stroke. The PT lesion and behavioural deficits were similar to the above (Fig. 5A and Supplementary Fig. 2A). Functional network organization was evaluated via RSFC mapping (i.e. zero-lag correlation of infraslow activity between 0.009 and 0.08 Hz) for all pairwise comparisons within our field of view (Fig. 5B–D). Group-averaged whole cortex correlation matrices were organized by functional assignment, then hemisphere (L: left, ipsilesional; R: right contralesional). Absent stroke, Vehicle (Fig. 5B) and MTEP-treated (Supplementary Fig. 2B) groups exhibited robust patterns of RSFC that feature prominently in healthy mice, including strong positive (reds) ipsilateral RSFC within each network (along main diagonal), as well as mirrored homotopic RSFC contralateral to each network (off diagonal elements). Additionally, strong anticorrelations (blues) were present between opposed functional networks, e.g. amongst sensorimotor and retrosplenial cortices. MTEP treatment in sham mice did not significantly affect RSFC (Supplementary Fig. 2B–D).

Following PT-stroke, RSFC disruption was observed at 14 days post-stroke in both Vehicle-treated (Fig. 5C) and MTEP-treated (Fig. 5D) groups primarily observed within ipsilesional (left) motor and somatosensory cortices. In line with previous work,^29^ we hypothesized that improved sensorimotor function induced by MTEP would be associated with increased RSFC within and across regions spared from direct injury. To examine the effect of MTEP treatment on global patterns of RSFC after PT, group-wise correlation differences were calculated as MTEP post-PT minus Vehicle post-PT (Fig. 5E).

After stroke, MTEP-treated mice exhibited higher intrahemispheric RSFC within contralesional somatosensory, motor and surrounding cortices [Fig. 5E (box 1)], ipsilesional [Fig. 5E (box 2)] and contralesional [Fig. 5E (box 3)] visual cortices, as well as stronger homotopic RSFC in visual regions compared with Vehicle-treated mice [Fig. 5E (box 4)]. Furthermore, larger anticorrelations between anterior-posterior brain regions were more pronounced in MTEP-treated mice compared with Vehicle-treated mice [Fig. 5E (box 5)].

The most salient RSFC differences across groups were evaluated using unbiased spatial PCA performed on the group-level correlation difference matrix (Fig. 5F). PC1 explained 53% of the inter-group variance at 14 days after stroke and was determined significant through extensive permutation resampling (*P* = 0.026). A map of PC1 (Fig. 5G) revealed the topography associated with these group-wise differences and included nearly all of the motor and somatosensory cortex (reds) in the contralesional hemisphere and large correlation differences between visual/retrosplenial regions and anterior sensorimotor regions (blues). These findings were not dependent on whether group-wise lesions were included or excluded in the PCA (Supplementary Fig. 3).

### MTEP treatment after stroke results in functional network topology similar to healthy mice

Normal functional brain organization is characterized by a balance of network segregation and integration which optimizes local processing and global information transfer.^62^ Given that MTEP-treated mice exhibited significantly better behavioural performance compared with Vehicle-treated mice, we hypothesized that MTEP-treatment after stroke would result in a return to more normalized network topology compared with Vehicle treatment after stroke.

**Figure 6:**
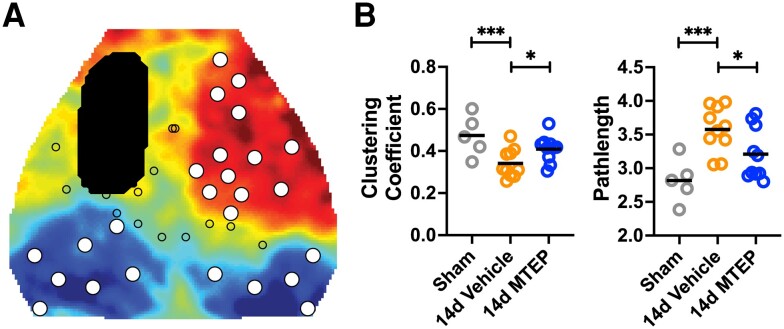
**MTEP treatment results in functional brain topology comparable to pre-stroke organization**. (**A**) Map of principal component 1 (PC1) from Fig. 5G with overlays of the photothrombotic (PT) infarct (black oval) and functional network nodes in our field of view. Node locations (all circles) were determined by the centre of mass of each functional region as per the Paxinos atlas in Fig. 5A. Larger white filled circles indicate brain nodes used for graph analysis, and determined by being outside of direct injury and within the top 30th percentile of all pixels in PC1 (i.e. to select for only those nodes associated with the largest group-wise differences). (**B**) Local and global functional network topology was assessed through graph measures of the weighted, undirected clustering coefficient and shortest path length across nodes. Functional network properties were averaged across nodes to get a local and global measure of brain organization for each mouse in each group. Following stroke, Vehicle-treated mice exhibited significant disruption in clustering coefficient (*P* < 0.01) and average path length (*P* < 0.001) compared with controls. Conversely, MTEP-treated mice exhibited higher clustering coefficient (*P* < 0.05) and lower path length (*P* < 0.05) compared to Vehicle-treated mice and had a clustering coefficient (*P* > 0.05) and path length (*P* > 0.05) indistinguishable from sham mice (one-way ANOVA, followed by false discovery rate correction). d = days.

**Figure 7:**
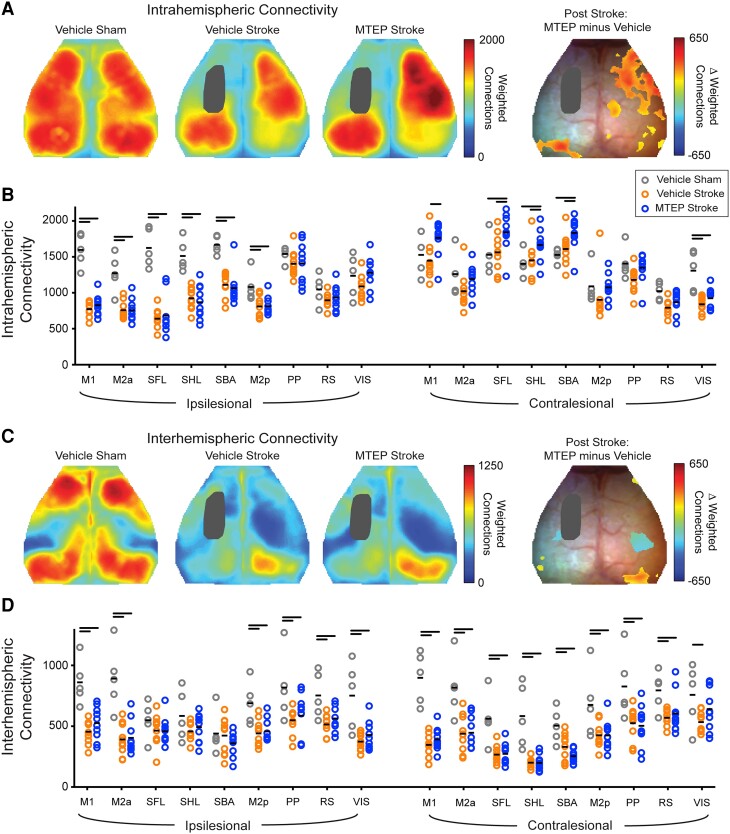
**Inhibition of mGluR5 increases intra- and interhemispheric functional connection density.** Intrahemispheric node degree was quantified for each pixel as the number of functional connections within the ipsilateral hemisphere having a positive correlation coefficient [*z*(r) > 0] (see text). (**A**) Group-averaged maps of weighted intrahemispheric node degree in the Vehicle sham and stroke groups (Vehicle stroke and MTEP stroke) 14 days after photothrombotic (PT)-stroke. Prior to stroke high node degree is observed over both hemispheres, with higher intrahemispheric connectivity (reds) within somatosensory and motor cortices, and parts of visual cortex. Following stroke, large reductions in ipsilateral node degree are clearly observed around the infarct core in regions surrounding the infarct in both groups. Difference maps reveals that MTEP treatment after PT-stroke resulted in significantly increased node degree over large portions of contralateral motor and sensory regions compared to sham and Vehicle. (**B**) Quantification of node degree in regions defined by atlas assignments in the Vehicle sham and stroke groups. Compared to Vehicle-treated sham, stroke Vehicle mice exhibited reduced intrahemispheric node degree in peri-lesional regions [primary motor (M1); anterior posterior secondary motor (M2a); somatosensory forelimb (SFL); somatosensory hindlimb (SHL); posterior secondary motor (M2p)]. Treatment with MTEP after stroke significantly increased intrahemispheric node degree in contralesional primary motor and somatosensory regions compared with Vehicle. Horizontal lines above bars indicate significant differences, *P* < 0.05 (two-way ANOVA, followed by false discovery rate correction). Data are available in Supplementary Table 1. (**C**) Intrahemispheric node degree was quantified for each pixel as the number of functional connections within the contralateral hemisphere having a positive correlation coefficient [*z*(r) > 0] (see text). Cortical disruptions in interhemispheric node degree were largely similar across treatment groups. However, Vehicle-treated mice exhibited higher interhemispheric node degree in right parietal and posterior somatosensory regions, while in the left visual cortex, significantly higher node-degree was observed in the MTEP group statistically indistinguishable from sham animals. (**D**) Quantification of node degree in regions defined by atlas assignments in the Vehicle sham and stroke groups. Compared with Vehicle-treated sham, stroke Vehicle mice exhibited reduced interhemispheric node degree across both hemispheres. There was no difference in interhemispheric node degree between the stroke groups. Horizontal bars indicate significant differences, *P* < 0.05; two-way ANOVA, followed by false discovery rate correction. Data are available in Supplementary Table 2. M1 = primary motor; M2a = anterior posterior secondary motor; SFL = somatosensory forelimb; SHL = somatosensory hindlimb; SBA = somatosensory barrel; M2p = posterior secondary motor; PP = posterior parietal; RS = retrosplenial; VIS = visual cortex.

Local and global functional network organization was assessed through the weighted undirected clustering coefficient, shortest path length (Fig. 6) and node degree (Fig. 7). The clustering coefficient and shortest path length were determined for functional network nodes defined by stereotactic location, which overlapped with the largest group-level RSFC differences (white circles, Fig. 6A) observed across MTEP- and Vehicle-treated groups after stroke.

Following stroke, Vehicle-treated mice exhibited significant disruption in clustering coefficient (*P* < 0.001) and average path length (*P* < 0.001) compared with the controls (Fig. 6B). Conversely, MTEP-treated mice exhibited higher clustering coefficient (*P* < 0.05) and lower path length (*P* < 0.05) compared with Vehicle-treated mice and had a clustering coefficient (*P* > 0.05) and path length (*P* > 0.05) indistinguishable from sham mice (Fig. 6B).

Intrahemispheric functional connection density was quantified for each pixel as the number of functional connections within each hemisphere having a positive correlation coefficient (Fig. 7). Prior to stroke, high node degree was observed over both hemispheres, with higher intrahemispheric connectivity (reds) within somatosensory and motor cortices, and parts of visual cortex, with fewer connections (greens, blues) exhibited by medial regions (Vehicle Sham, Fig. 7A and B). Intrahemispheric node degree was similar between Vehicle- and MTEP-treated sham groups (Supplementary Fig. 2C). Following stroke, large reductions in node degree were clearly observed in regions surrounding the infarct in both groups (Vehicle Stroke and MTEP Stroke, Fig. 7A, and grey and blue bars, Fig. 7B). Interestingly, stroke also resulted in significant reductions in node degree in contralesional visual cortex (*P* < 0.001; Fig. 7B). Unlike Vehicle, MTEP treatment after PT resulted in significantly increased node degree over large portions of contralateral motor and sensory regions compared with sham and Vehicle (difference map, Fig. 7A and B; quantified in Supplementary Table 1). These findings were not dependent on whether group-wise lesions were included or excluded in the intrahemispheric node degree analysis (Supplementary Fig. 4A).

Similarly, interhemispheric node degree was assessed for each pixel as the number of positive functional connections within the contralateral hemisphere (Fig. 7C and D). Stroke reduced interhemispheric node degree globally over the cortex in both groups (Fig. 7D and quantified in Supplementary Table 2). Cortical disruptions in interhemispheric node degree were largely similar across groups. However, in contralesional visual cortex, Vehicle-treated mice exhibited significantly lower interhemispheric node degree, while the MTEP group was statistically indistinguishable from sham animals (Fig. 7D). Interhemispheric node degree was similar between Vehicle- and MTEP-treated sham groups (Supplementary Fig. 2D). These findings were not dependent on whether group-wise lesions were included or excluded in the interhemispheric node degree analysis (Supplementary Fig. 4B).

Taken together, stroke results in functional disconnections between ipsi- and contra-lesional sensorimotor cortex, and distant regions including ipsi- and contralesional visual cortices. Connectomal diaschisis within contralesional sensorimotor and bilateral visual cortices observed in Vehicle-treated mice was reversed following 12 days of MTEP treatment. In addition to observing RSFC reconnections, local and global functional brain organization in MTEP-treated mice exhibited network topologies akin to the uninjured brain, consistent with the improved functional recovery observed in mice receiving MTEP.

### Increased RSFC in contralateral sensorimotor cortex is not due to changes in tissue levels of mGluR5

**Figure 8:**
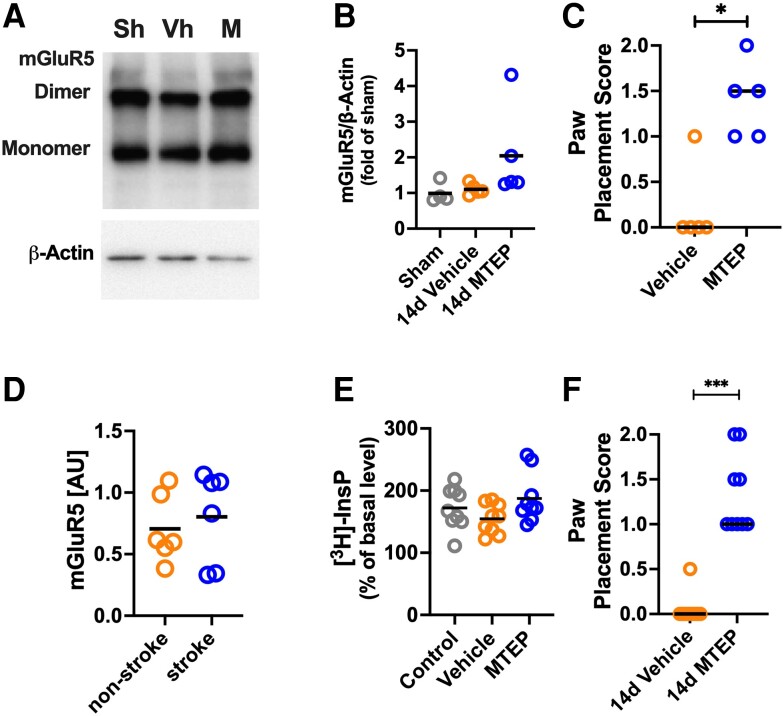
**Increased functional connectivity in contralateral sensorimotor cortex after inhibition of mGluR5 is not associated with changes in tissue levels of mGluR5**. (**A**) Levels of mGluR5 in sensorimotor cortex analysed by western blotting in (**B**) Sham group (*n* = 4) and mice subjected to photothrombotic (PT)-stroke and 14 days of recovery and treated with Vehicle (*n* = 5) and MTEP (*n* = 5). No significant differences were found among groups [one-way analysis of variance (ANOVA); bar denotes mean]. (**C**) Paw placement score of mice used in **B** [Kruskal–Wallis test with a *post hoc* Dunn’s multiple comparison (MC) test; *P < 0.05; bar denotes median]. (**D**) Homogenates were generated from cortex of the contralateral hemisphere from deceased stroke patients and non-stroke subjects (for details see Supplementary Table 3). There is no difference in the levels of mGluR5 between the groups (one-way ANOVA; bar denotes mean). (**E**) Formation of mGluR5-mediated inositol phosphate [^3^H]InsP in brain slices prepared from the contralateral sensorimotor cortex in control mice (*n* = 9) and mice after PT-stroke and 14 days of recovery and treated with Vehicle (*n* = 9) or MTEP (*n* = 9). No significant differences were found among groups (one-way ANOVA; bar denotes mean). (**F**) Paw placement score of mice used in **E** (Kruskal–Wallis test with a *post hoc* Dunn’s MC test; ***P < 0.001; bar denotes median). d = days; M = MTEP; Sh = Sham; Vh = Vehicle.

To explain the restorative effect of mGluR5 NAMs on sensorimotor function, we examined post-stroke mGluR5 in the contralateral sensorimotor cortex. There were no differences in mGluR5 protein levels in contralateral sensorimotor cortices among the sham-operated, Vehicle-treated and MTEP-treated groups at 14 days of recovery (Fig. 8A and B). The PP score improved in these MTEP-treated mice (Fig. 8C; *P* < 0.001).

As a translational correlate, and to assess whether the target for mGluR5 NAMs is present in the contralateral cortex of stroke patients, we measured the levels of mGluR5 in brain tissue from deceased patients. We found no difference in mGluR5 levels in the contralateral brain tissue between the stroke patient group and a non-stroke group (Fig. 8D and Supplementary Table 3).

The maximal agonist-stimulated effect on mGluR5-mediated signal transduction was measured as [^3^H]InsP accumulated in slices prepared from the contralateral sensorimotor cortex in response to the mGlu1/5 receptor agonist DHPG and in the presence of the mGluR1 inhibitor JNJ1625968. There was no difference in [^3^H]InsP formation among sham-operated, Vehicle-treated or MTEP-treated mice at 14 days after stroke (Fig. 8E). The PP score improved in these MTEP-treated mice (Fig. 8F; *P* < 001).

A shift in neuronal excitability due to changes in GABAergic activity, in particular parvalbumin (PV)-expressing GABA neurons (Supplementary Fig. 5A–D), could influence brain connectivity.^63^ The levels of the GABA synthesizing enzymes GAD65 (Supplementary Fig. 5E), GAD67 (Supplementary Fig. 5F), as well as PV (Supplementary Fig. 5G) were assessed by western blots in tissue homogenates from contralateral primary sensorimotor cortex in sham-operated mice and 14 days after PT in mice treated with Vehicle or MTEP. There were no significant changes in the levels of GAD65, GAD67 or PV among the experimental groups.

We previously reported that increased contralesional motor RSFC was inversely correlated with the number of GABA/PV neurons.^29^ We therefore assessed the number of PV immunoreactive neurons in the secondary motor area (M2), the primary motor area (M1) and a somatosensory area (SS) of the contralateral cortex of the mouse brain at 14 days of recovery (Supplementary Fig. 5H and I). There was no difference in the number of PV immunoreactive cells among the sham group and the Vehicle- and MTEP-treated groups in any region examined (Supplementary Fig. 5I).

The PV/GABA neurons in cortex are enwrapped by chondroitin sulphate containing proteoglycans (CSPG)-containing perineuronal nets (Supplementary Fig. 5J). We counted the number of perineuronal nets in the contralateral mouse somatosensory cortex of the mouse brain 14 days after stroke. There were no differences in the Vehicle- and MTEP-treated groups (Supplementary Fig. 5K)

## Discussion

The lack of post-acute restorative stroke treatments has prompted a search for novel recovery enhancing interventions.^5–7^ We present evidence that inhibition of mGluR5 with small molecular weight compounds restores brain connectivity and sensorimotor functions after stroke. These compounds, in clinical trials for other diseases, potentially present a novel pharmacological therapy after stroke to facilitate patient rehabilitation.

We observed that mGluR5 NAMs administered in the post-acute phase after stroke markedly restored tactile/proprioceptive pawplacement functions in a manner similar to, and reinforced by, multisensory stimulation. The benefits of the treatment spanned across species and gender and was effective in animals subjected to cortical (PT) and mixed cortical and subcortical (MCAO) stroke lesions. We determined that the therapeutic effect was mediated by mGluR5 inhibition, since pretreatment with the mGluR5 PAM VU0360172 prevented recovery, and mice lacking mGluR5 regained PP performance in similar manner to wild-type mice with mGluR5 activity blocked by MTEP. These effects are not specific to MTEP but were also observed following treatment with AFQ075 or fenobam, two mGluR5 NAMs with molecular structures distinct from MTEP.

### Recovery enhancement of sensorimotor functions by mGluR5 NAMs is not due to neuroprotection

Since allosteric mGluR5 modulators are neuroprotective when given within 3 h after experimental injury,^64–66^ the processes underlying behavioural improvements could reside in the protected tissue, in addition to plasticity in the tissue remote from injury. Here we show for the first time, and unequivocally, that treatment with mGluR5 NAMs starting 2–10 days after stroke, i.e. outside the neuroprotective therapeutic time window,^67^ enhances recovery of sensorimotor functions without affecting infarct size, implying that processes outside of the matured infarct facilitate recovery.

### Transient and persistent recovery responses to mGluR5 inhibition

The treatment response has two components: a rapid, transient response, followed by a slower, more persistent change. The transient PP response to the first MTEP treatment at 2 days after stroke is in line with the rapid peak in MTEP plasma levels.^41^ The rapid response to MTEP delivery suggests that existing neuronal circuitry subserving sensorimotor function in tissue surrounding and remote from the infarct is depressed by an mGluR5-dependent mechanism leading to ‘permanent diaschisis’ after stroke.^12^ Subacute suppression of mGluR5 activity appears to relieve the negative influence of mGluR5 on circuit level communication, and when continued over weeks, enhances plasticity processes that permanently alleviate the inhibitory action of mGluR5 on recovery. A similar transient and subsequent progressive recovery response was reported after amphetamine treatment following brain injury in rats and cats.^68,69^ Moreover, the required 12 days inhibition of mGluR5 is consistent with the reported critical period for recovery promoting interventions after stroke.^70–72^

Recovery is evident in multiple behavioural sensorimotor tests and particularly apparent in the tactile/proprioceptive PP test. Enriched housing after stroke optimizes recovery of sensorimotor functions^27^ and produces similar performance enhancements to those observed following mGluR5 inhibition. Interestingly, activation of mGluR5 by VU0360172 while mice were in enriched housing, prevented recovery, indicating that mGluR5 has an integrated role in EE-mediated mechanisms of recovery. Concurrently treating mice with a suboptimal recovery-enhancing dose of MTEP (1 mg/kg), which provides approximately 50% receptor occupancy,^42^ while housed in an EE, had an additive recovery-enhancing effect. Together, these data indicate that mGluR5 inhibition and multimodal stimulation of the brain by an EE target similar or overlapping cellular recovery-promoting mechanisms. From a clinical perspective, the additive effect of MTEP treatment and enriched housing suggests that combined treatment of mGluR5 NAMs and early rehabilitative training will act in concert to promote recovery.

### mGluR5 NAMs restore brain connectivity after stroke

Recovery enhancements were associated with restoration of functional brain organization in the surviving tissue assessed with optical intrinsic signal imaging. Loss of RSFC was observed following PT stroke among regions remote from the lesion, including ipsi- and contralesional visual cortices, and sensorimotor areas of the contralesional hemisphere consistent with our prior work.^29^ Behavioural recovery following 12 days of MTEP treatment improved intrahemispheric RSFC in several brain regions including contralesional sensorimotor cortex and visual cortices of both hemispheres. Brain networks returning towards more normal patterns of intrinsic organization after stroke appear to support better behavioural performance.^19,20^ We have recently shown that improved tactile proprioception after stroke is associated with increased node degree in several brain regions relevant for processing proprioception and touch.^29^ Furthermore, chronic increases in contralesional excitatory activity suppress global network interactions and local remapping of lesioned tissue.^57^ In the present study, the patterns of RSFC in MTEP-treated mice appeared to reflect the alleviated connectomal diaschisis observed in Vehicle-treated animals and represent brain network topologies akin to the uninjured brain, consistent with findings in stroke patients.^73^ Importantly, including or excluding lesioned tissue from the RSFC analysis did not significantly alter these observations.

RSFC recovery in the contralesional sensorimotor cortex is a common feature after both MTEP treatment and exposure to EEs,^29^ strongly suggesting a central involvement of this region in post-stroke somatosensory processing.^23,74^ In the setting of an EE, inhibition of the contralesional cortex in rats exposed to enriched-rehabilitation for 4 weeks following MCAO induced greater deficits in paretic limb function compared with control animals or rats recovering from small lesions.^23^ Furthermore, environmental enrichment after stroke increases contralateral spine density of layer II-III pyramidal neurons,^30^ while treatment with Nogo A antibodies provides recovery of function in rats with large stroke lesions dependent on plasticity processes in the contralateral cortex.^75^

Recovery after stroke requires an anatomical substrate to facilitate remodelling. Recovery in the context of smaller ischaemic lesions is associated with remapping and reorganization in functionally-related areas adjacent to the lesion.^76^ Inactivation of the contralesional hemisphere appears to favour spontaneous recovery of the impaired forelimb in rats.^77^ Conversely, increased activity of the unaffected limb impairs recovery processes in mice.^57,78^ In the clinical setting, patients exhibiting smaller lesion sizes respond well to interventional strategies suppressing contralesional motor cortex.^79^ Recovery associated with larger lesion sizes appears to depend on the recruitment of more distant and contralesional areas,^79–81^ possibly due to low structural reserves in tissue adjacent to the lesion. Patients with poor upper extremity function report clinical improvements after 12 weeks of training, results associated with increased activation in bilateral premotor and contralesional sensorimotor corticies.^82^ Additionally, in patients with severe stroke deficits, gradually increasing activity in contralesional motor cortices correlates with improved functional outcome.^21^ Conversely, suppression of contralesional M1 excitability, using transcranial direct current stimulation, leads to increased dysfunction in patients recovering from moderate to severe impairments.^83^ Similar findings have been reported in patients recovering from subcortical stroke and severe deficits; suppressing contralesional M1 or premotor activity using transcranial magnetic stimulation deteriorated motor performance. Collectively, these findings suggest a supportive role of contralesional tissue in patients recovering from large lesions.^84^

Optical intrinsic signal imaging allows for mapping relationships between reorganization of cortical functional circuits and recovery. However, subcortical structures such as the striatum and thalamus^85,86^ could also play a role in information transfer to and from somatosensory cortex, which could depend on mGluR5. For example, in a mouse model of FXS, the mGluR5 NAM AFQ056 partially restored the aberrant brain connectivity between somatosensory areas and the striatum.^37^

In our study, the observed loss of function after stroke was not due to increased levels of mGluR5, unlike prior reports in, for example, experimental Parkinson’s disease-levodopa-induced dyskinesia.^36^ Instead, we envisage that mGluR5 contributes to downscaling of synaptic activity^33^ leading to persistent synaptic depression potentially through a long-term depression-like mechanism.^34^ The maladaptive plasticity mediated by mGluR5 could be due to an imbalance of excitation-inhibition after stroke contributed by GABAergic interneurons.^29,70^ We demonstrated that EE housing reduced the number of immunoreactive neurons in the contralateral motor cortex which correlated with increased RSFC.^29^ Here, the number of PV+ neurons in the contralateral cortex of Vehicle- and MTEP-treated mice were unaltered, as were levels of the GABA synthesizing enzymes GAD65 or GAD67, or PV. The GABA/PV cells are enwrapped with CSPG containing perineuronal nets that are important regulators of plasticity in the healthy brain^87^ and possibly following stroke.^27^ However, we did not find any changes in the number of perineuronal nets in the contralateral hemisphere after MTEP treatment.

Several other mGluR5 regulated mechanisms could potentially modulate recovery after stroke,^16,88^ such as changes in growth factor levels including BDNF,^71,89^ brain inflammation^35^ or axogenesis.^90,91^ Further research is needed to understand the mechanisms underlying the enhancement of recovery by mGluR5 NAMs after stroke.

### Clinical implications

Importantly, because we show that mGluR5 is present in relevant regions of injured brain tissue in stroke patients, they may be amenable to therapeutic interventions. Still, outside of stroke, the clinical effects of mGluR5 NAMs in other brain disorders depend on context, dose and timing,^35^ which has implications for the clinical trial design of these compounds in stroke recovery. Translating preclinical findings into successful clinical trials has generally been unsuccessful. This failure might be due to patient stratification, monitoring irrelevant outcome measures or incomplete attention to the design of preclinical experiments and associated findings. With regard to these points, our preclinical data suggest that treatment with mGluR5 NAMs would be applicable to patients with moderate to severe stroke presenting with deficits in touch and proprioception. Treatment should be initiated in the post-acute phase, last for at least 2 weeks, and its efficacy assessed with a primary outcome measure of tactile and proprioceptive functions of somatosensation.^92,93^ Further, for optimal clinical effect, mGluR5 NAMs should be combined with relevant rehabilitative training paradigms.^28,75^

## Supplementary Material

awad293_Supplementary_Data

## Data Availability

The data are available from the corresponding authors upon reasonable request.
