# Supplementary material for: Inhibiting metabotropic glutamate receptor 5 after stroke restores brain function and connectivity
Source: Brain. 2023 Sep 1;147(1):186–200. doi: 10.1093/brain/awad293 (PMC10766240; doi:10.1093/brain/awad293)
Supplement: awad293_Supplementary_Data [file awad293_supplementary_data.pdf]

Supplementary Material

Supplementary Figure 1

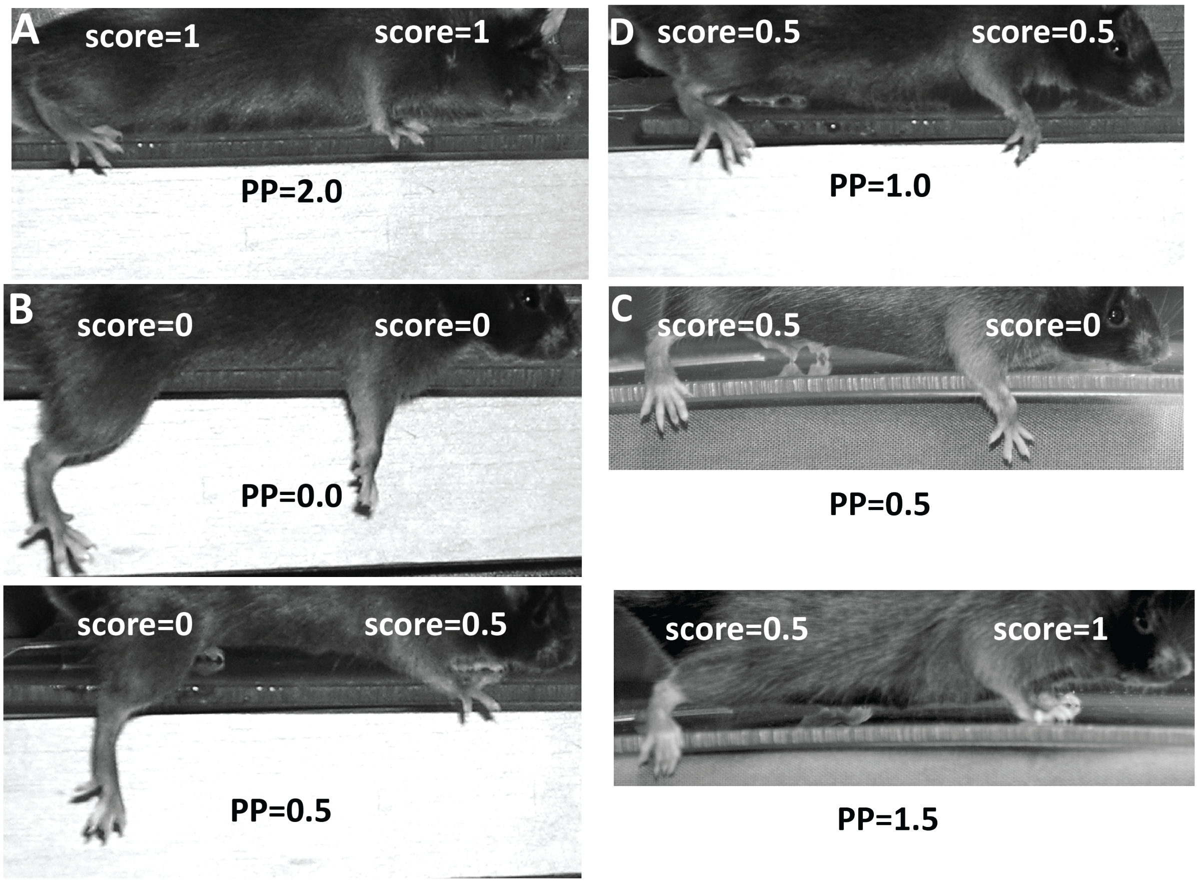

Supplementary Figure 1 The Paw Placement (PP) test. Animals are placed on a platform and moved laterally until the two paws loose contact with the platform and proprioceptive sensory stimulation. Tactile stimulation is then provided by moving the animal back to the edge of the platform and the movement, posture and placement of limbs, paws and digits assessed. A score 1, 0.5 or 0 for each paw is noted and the sum of the scores registered as a paw placement (PP) score. **(A)** score 1: Fast placement of the limb and paw onto the table; **(B)** Score 0: limb, paws and digits extended; **(C-F)** Score 0.5: incomplete placing of the limb with extended digits or supinated paw inwards towards the edge.

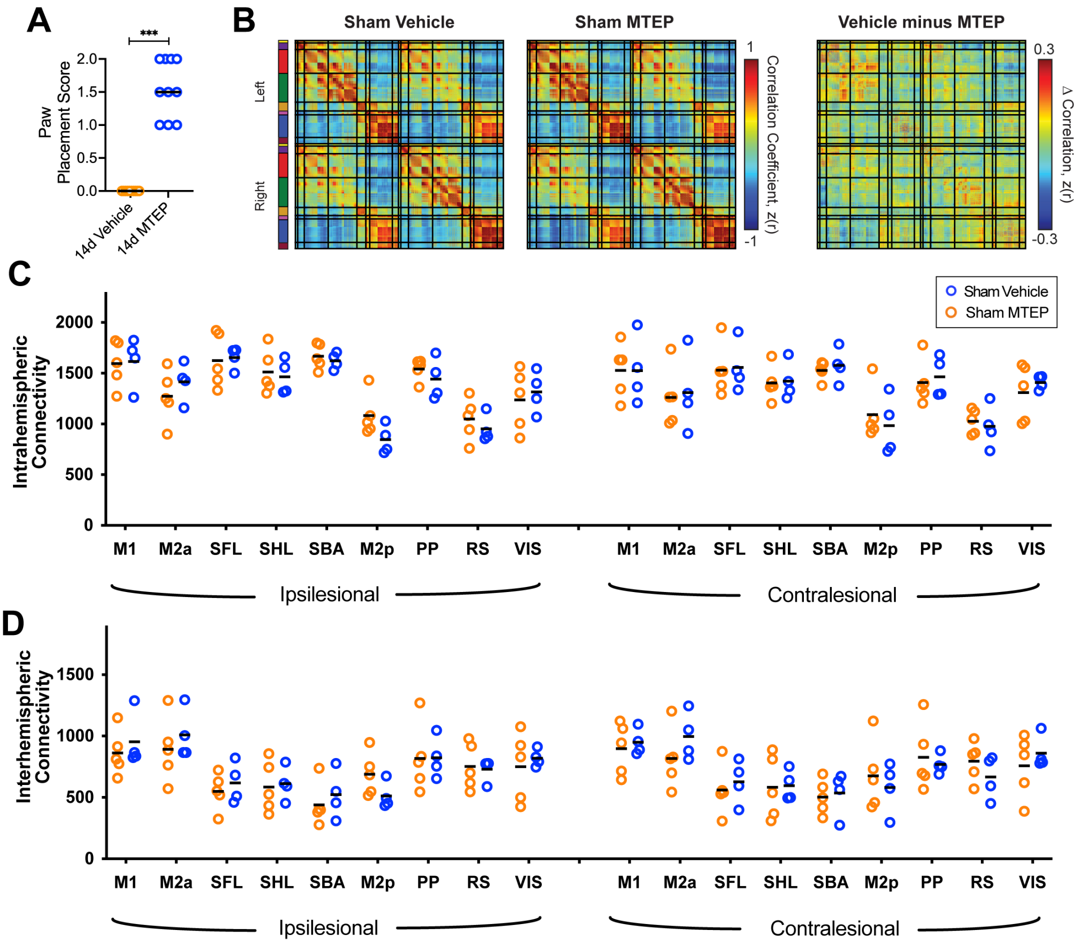

Supplementary Figure 2 (A) Paw placement score of mice generating the OIS data set. All mice had a score of 2 prior to stroke and a score 0 at day 2 after stroke prior start of daily treatment with Vehicle (*n*=10) or MTEP (5mg/kg i.p., *n*=10) (Mann-Whitney test, ***p<0.001. Bar denotes median). **(B)** Group-averaged, whole-cortex correlation matrices for sham vehicle (left) and (middle) sham MTEP treatment. (right) Correlation differences matrix calculated as Sham Vehicle minus Sham MTEP. Matrices are grouped by functional assignment (see Fig. 5 for network legend) and organized by hemisphere (left, ipsilesional; right contralesional). **(C)** Intrahemispheric node degree and **(D)** Interhemispheric node degree was quantified for Sham groups in regions defined by atlas assignments. There was no difference between vehicle treated sham and MTEP treated sham mice (t-test, followed by FDR correction for multiple comparisons.

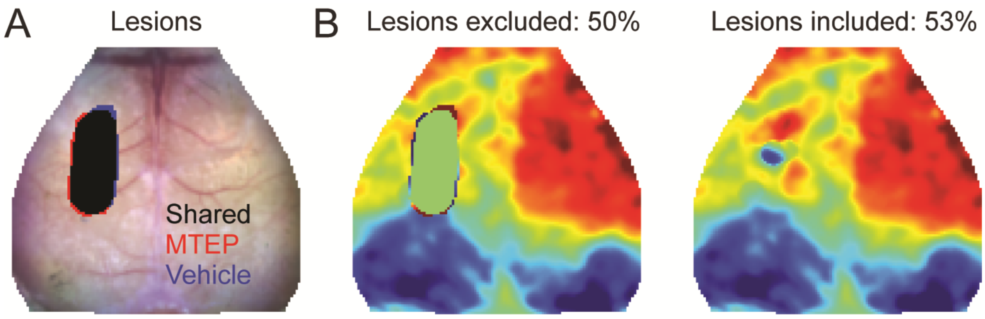

Supplementary Figure 3 The effects of MTEP on global RSFC does not depend on whether lesioned pixels are included in the RSFC analysis. (A) Lesion map for each group. Group-wise lesions were defined as any pixel having a lesion incidence greater than 50% for that group. Lesions in MTEP (Red) and Vehicle (Blue) treated mice overlap by 92% (black), with lesion borders within 80 microns of one another. **(B)** Group-wise differences in whole cortex RSFC (i.e., all pairwise correlations within our field of view) were evaluated by applying spatial PCA to the whole-cortex RSFC difference matrix with and without lesioned pixels included. As described in the main text, the first principle component (PC1) reports the topography associated with the largest group-wise differences in RSFC structure (B, right panels). Including or excluding lesioned pixels does not appreciably alter the topography of PC1 nor the variance these PCs explain (Lesion pixels included: 53%; Lesion pixels excluded: 50%). In either case, mice treated with MTEP exhibit larger changes in RSFC within contralesional motor and sensory cortex, as well as ipsilateral parietal and visual cortex and contralesional posterior visual cortex. Not surprisingly minor differences between the maps occur around the lesion itself and could possibly account for the small difference in variance explained by PC1 alone.

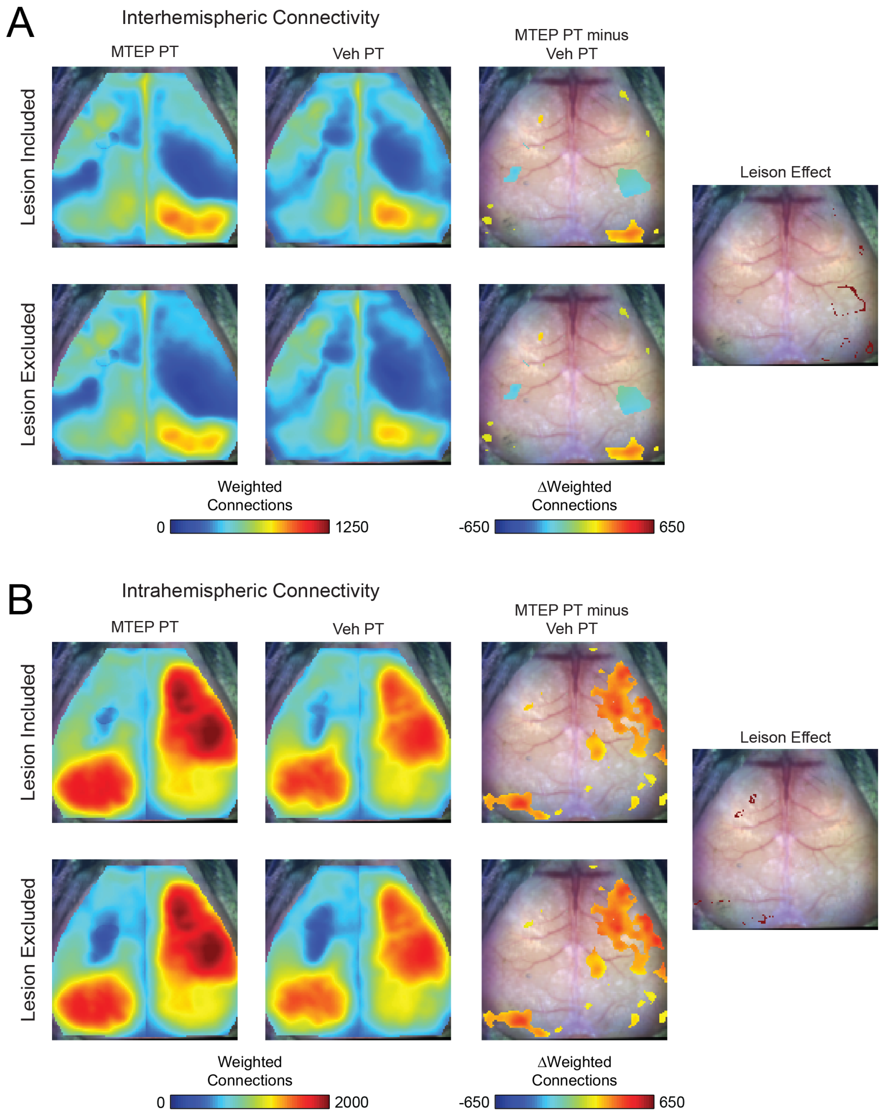

Supplementary Figure 4 The effects of MTEP on functional connection density do not depend on whether lesioned pixels are included in the analysis. Weighted node degree was calculated for all pixels having a correlation coefficient > 0 with any pixel in the **(A)** ipsilateral (intra) or **(B)** contralateral (inter) hemisphere of a candidate pixel. Compared to ND calculations including lesioned pixels, modest reductions in overall ND strength were observed in both groups when lesioned tissue was excluded from analysis (bottom rows), most evident in interhemispheric ND measures. This result is expect as fewer candidate pixels are allowed in the ND calculation when excluding lesioned pixels. However, maps of group-wise differences in ND (corrected $p<0.05$) were not significantly impacted by inclusion/exclusion of the lesion and reveal near-identical regional differences. A “Lesion Effect” was defined as any significantly different brain pixel not shared across the lesion included/excluded ND difference maps. For intra hemispheric ND, this analysis reveals a small number of pixels surrounding the large cluster observed in both analyses within lateral parietal and primary somatosensory

barrel cortex, or scattered pixels in other brain regions (52 pixels total out of ~11000). Similarly, including lesioned tissue in the interhemispheric ND measure affects 88 pixels out of ~11000, most of which are surrounding the lesion or scattered throughout small portions of visual cortex.

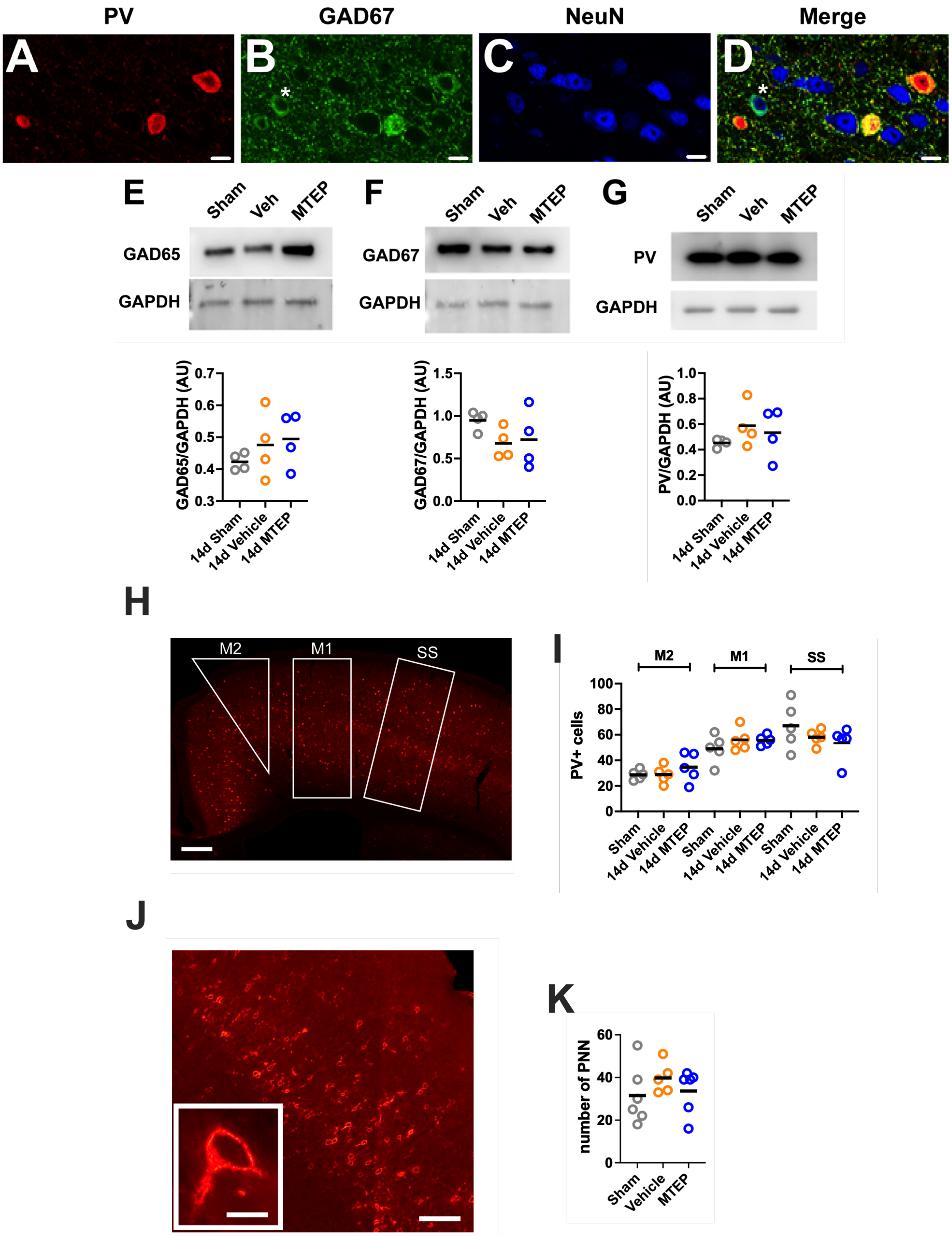

Supplementary Figure 5 Inhibition of mGluR5 did not change GAD65, GAD 67, Parvalbumin or Perineuronal Nets in the contralateral somatosensory cortex after stroke. Confocal immunofluorescence micrograph of the mouse in somatosensory cortex displaying **(A)** Parvalbumin immunopositive neurons (red), **(B)** GAD67 immunopositive (GAD67+) neurons (green), **(C)** NeuN immunopositive (NeuN+) neurons (blue), and **(D)** the merged micrograph. GAD67/PV neurons appear orange, (*) indicates a GAD67 neuron lacking

PV. **(E)** Levels of the GABA synthesizing enzymes GAD65, **(F)** GAD67 and **(G)** parvalbumin (PV), in tissue homogenates of contralateral somatosensory cortex in sham-operated mice (*n*=4) and mice after PT-stroke and 14 days of recovery and treated with vehicle (n=4) and MTEP (*n*=4). No significant difference between groups was found by analysis of variance. Bar denotes mean. Full-length gels are displayed in Supplementary Figure 6. **(H)** Immunofluorescence photomicrograph of the mouse brain cortex contralateral to injury with parvalbumin immunopositive (PV+) cell bodies (red) and with brain areas of cell counts depicted. Bar 200um. **(I)** The number of PV+ cells in the contralateral secondary motor cortex (M2), the primary motor cortex (M1) and the somatosensory cortex (SS) of the mouse brain as indicated in K, of Sham (*n*=5) and PT mice at 14 days recovery, treated with Vehicle (*n*=5) or MTEP (*n*=5). No significant differences were found among groups in each region was found by analysis of variance. Bar denotes mean. **(K)** Immunofluorescence photomicrograph of the rat somatosensory with perineuronal net structures (PNNs, red). Bar 200um, insert bar 20um. **(L)** The number of PNNs in the contralateral somatosensory cortex as shown in M of sham-operated (n=6), Vehicle (n=5) and MTEP treated rats at 14 after stroke. No significant difference between groups was found by analysis of variance. Bar denotes mean.

Supplementary Table 1 Quantification of intrahemispheric node degree connectivity 14 days after sham or photothrombotic stroke.

Region	Sham Veh (A)	Sham MTEP (B)	Stroke Veh (C)	Stroke MTEP (D)	A vs C	A vs D	C vs D
**M1-l**	1597±229	1616±246	774±101	828±138	<0.001	<0.001	0,549
**M1-r**	1529±266	1527±332	1448±264	1765±240	0,465	0,051	0,002
**M2a-l**	1274±258	1415±191	760±115	757±153	<0.001	<0.001	0,975
**M2a-r**	1263±293	1311±383	1023±248	1192±198	0,093	0,525	0,093
**SFL-l**	1625±268	1654=106	641±130	685±273	<0.001	<0.001	0,627
**SFL-r**	1531±253	1558±247	1566±259	1843±260	0,756	0,008	0,007
**SHL**	1513±218	1465±172	926±151	869±229	<0.001	<0.001	0,526
**SHL-r**	1405±169	1423±188	1453±230	1667±212	0,66	0,028	0,028
**SBA-l**	1668±123	1623±80	1112±163	1065±226	<0.001	<0.001	0,597
**SBA-r**	1528±91	1577±168	1611±216	1836±223	0,454	0,017	0,02
**M2p-l**	1082±204	846±142	811±140	816±123	0,025	0,025	0,956
**M2p-r**	1091±259	983±290	904±331	1075±173	0,138	0,885	0,138
**PP-l**	1542±105	1442±204	1406±178	1410±287	0,352	0,352	0,970
**PP-r**	1408±219	1466±202	1182±176	1350±197	0,096	0,596	0,096
**RS-l**	1048±206	950±135	897±102	934±164	0,454	0,454	0,686
**RS-r**	1025±122	975±214	793±105	871±147	0,110	0,248	0,388
**VIS-l**	1239±298	1317±205	1089±162	1280±217	0,266	0,711	0,107
**VIS-r**	1309±279	1409±68	842±100	925±139	<0.001	<0.001	0,359

Mean Intra-hemispheric Node Degree for individual regions, and differences between Sham Vehicle, stroke vehicle and Stroke MTEP groups. All node degree data were calculated with lesioned pixels included. Reported P-values are following a two-way ANOVA and FDR corrected. *Abbreviations:* M1: primary motor, M2: secondary motor, SFL: somatosensory forelimb, SHL: somatosensory hind limb, SBA: somatosensory barrel, M2p: posterior secondary motor, PP: posterior parietal, RS: retrosplenial, VIS: visual, -l: left injured hemisphere, -r: right contralesional hemisphere.

Supplementary Table 2 Quantification of interhemispheric node degree connectivity 14 days after sham or photothrombotic stroke.

Region	Sham Veh (A)	Sham MTEP (B)	Stroke Veh (C)	Stroke MTEP (D)	A vs C	A vs D	C vs D
**M1-l**	862±185	954±224	455±96	526±133	<0.001	<0.001	0,261
**M1-r**	898±211	950±107	346±90	392±92	<0.001	<0.001	0,468
**M2a-l**	892±265	1009±202	391±127	404±134	<0.001	<0.001	0,842
**M2a-r**	818±244	997±194	439±149	446±131	<0.001	<0.001	0,912
**SFL-l**	550±148	619±165	464±128	460±107	0,395	0,395	0,947
**SFL-r**	562±203	629±177	266±70	274±85	<0.001	<0.001	0,907
**SHL**	585±209	613±137	457±74	492±116	0,29	0,337	0,583
**SHL-r**	583±260	597±123	200±35	199±64	<0.001	<0.001	0,988
**SBA-l**	440±174	524±196	424±150	360±98	0,827	0,461	0,461
**SBA-r**	504±139	536±180	330±114	254±53	0,036	0,003	0,221
**M2p-l**	689±173	513±111	442±108	458±95	0,004	0,004	0,791
**M2p-r**	676±281	582±207	426±100	425±120	0,002	0,002	0,982
**PP-l**	819±277	823±167	549±107	586±178	0,001	0,004	0,555
**PP-r**	827±275	769±80	525±131	503±166	<0.001	<0.001	0,726
**RS-l**	753±188	730±93	515±78	564±102	0,006	0,021	0,435
**RS-r**	795±158	667±177	570±64	605±163	0,011	0,02	0,578
**VIS-l**	752±279	820±70	374±69	428±112	<0.001	<0.001	0,387
**VIS-r**	759±253	860±136	535±83	642±175	0,011	0,127	0,127

Mean Inter-hemispheric Node Degree for individual regions, and differences between Sham Vehicle, stroke vehicle and Stroke MTEP groups. All node degree data were calculated with lesioned pixels included. Reported P-values are following a two-way ANOVA and FDR corrected. *Abbreviations:* M1: primary motor, M2: secondary motor, SFL: somatosensory forelimb, SHL: somatosensory hind limb, SBA: somatosensory barrel, M2p: posterior secondary motor, PP: posterior parietal, RS: retrosplenial, VIS: visual, -l: left injured hemisphere, -r: right contralesional hemisphere.

Supplementary Table 3 Demographics of brain tissue from deceased patients for Western blot analysis.

Patient	Gender	Age	Cortical Region
Stroke	Male	87	Temporal
Stroke	Male	80	Occipital
Stroke	Female	52	Occipital
Stroke	Male	79	Frontal
Stroke	Male	68	Frontal
Stroke	Female	65	Frontal
Non-stroke	Female	86	Parietal
Non-stroke	Female	65	Parietal
Non-stroke	Male	83	Parietal
Non-stroke	Male	66	Frontal
Non-stroke	Female	68	Occipital
Non-stroke	Male	66	Frontal

Supplementary Table 4 Statistical tests.

Data Sets:

	**Parametric**	**Non-Parametric**
Behavior and Biochemistry	Infarct size<br>Adhesive removal test<br>Foot fault test<br>Grip force<br>Western blot<br>Cell counts<br>Inositol phosphate [3H]InsP	Paw placement test<br>Postural hang reflex test
Neuroimaging	Node degree, clustering coefficient, path length	Whole cortex RSFC

Statistical methods:

**Comparisons (Behavior and biochemistry)**	**Parametric Test**	**Non-parametric Test**
2 groups, unpaired	T-test	Mann Whitney’s U-test
2 groups, paired	T-test	Wilcoxon’s matched-paired signed test
3 or more groups	One-way ANOVA, post hoc 2 tailed T-tests, Sidak’s correction for multiple comparisons	Kruskal-Wallis test, post hoc Dunn’s test
**Comparisons (Neuroimaging)**		
2 groups, unpaired	T-test	Permutation resampling
3 or more groups	One-way ANOVA, post hoc 2 tailed T-tests, followed by FDR correction for multiple comparisons	
3 or more groups in multiple regions	Two-way ANOVA, post hoc 2 tailed T-tests, followed by FDR correction for multiple comparisons	

Supplementary Methods

Stroke models

The photothrombotic stroke

Mice were anesthetized with 2% isoflurane in O_2 under spontaneous respiration. The skull was exposed via a midline incision of the skin. A bolus of Rose Bengal (0.1 mg/kg, Sigma) was injected intravenously. Ten minutes after the injection, a 4 x 2 mm rectangular area (anterior-posterior AP = +2 to -2 mm and ML= 1 to 3mm) was illuminated in the left hemisphere for 20 min with a cold light source (3100K; Schott, KL 1500) with a green filter (Schott 515 nm P/N 258.314). The scalp was sutured, and the mice recovered. Sham surgeries were performed similarly but without illumination. Photothrombotic stroke in rats was performed with the following differences: 0.5ml Rose Bengal (10 mg/ml) was given and an area 8 x 4.5 mm (AP= +4 to -4 mm, ML= 0.5 to 5 mm) illuminated for 20 min.

The ET-1 MCAO model

For the endothelin-1 (ET-1) middle cerebral artery occlusion (MCAO) model, male Wistar rats were used. Stroke was induced by perivascular administration of the vasoconstrictor peptide endothelin-1 (Sigma Aldrich) (200 pmol in 3 µl of saline). Anesthesia was induced by a mixture of ketamine hydrochloride (60 mg/kg) and xylazine (12 mg/kg) injected intramuscularly, and rat placed in a stereotaxic frame received an intradermal injection of 0.2 ml of 2% lidocaine (Astra Pharma Inc.). A skin incision was made exposing the scull. A subdural guide cannula (8 mm length, 22-gauge stainless-steel needle (Braun Melungen AG Sterican^R)) was positioned in the left brain hemisphere (AP = +0.2 mm, ML= 5.2 mm). An injection cannula (27G, 0.4 x40 mm BL/LB, B. Braun Melungen AG Sterican^R) was positioned in the piriform cortex at dorsoventral coordinate DV= -8.0, approximately 0.5mm from MCA. The guide canula was secured with cement (Ketac^{TM} cement, 3M Deutschland GmbH). The scalp wound was sutured and rats singly housed recovered for one week (antibiotic Baytril 2.5%, 0.2 ml µl/kg s.c., diluted with saline 200 µl/rat and anti-inflammatory drug Voren, 0.1 ml/kg i.m.). Infusion of ET-1 was made manually by a 10 µl Hamilton syringe placed in the guide cannula and connected to the PE20 tubing with an infusion speed of ET-1 solution (1 µl/1 min). Control rats were infused with saline.

Behavioral tests

Beam Walk test

In the Beam Walk test, a sensorimotor test of walking and balance, a wooden beam (9 x 5.5mm and 1.5 m length) was mounted above the floor. A home cage was positioned at the end of the beam, and the rat trained to cross the beam over three consecutive days until it crossed the

beam with a maximal 1-2 foot slips. The performance was recorded with a video camera, and the total number of steps and faults for each paw counted. The mean fraction faults of the total number of steps was calculated from three tests.

Grid test

The grid test informs on gait including sensorimotor function and coordination. A wire net with openings of approximately 2 cm^2 was mounted on a frame with a mirror under the grid for foot fault registration by a video camera. Two Plexiglas walls 45 cm in length were placed on the net 8 cm apart forming a walkway. A home cage was placed at one end of the walkway. The mouse was trained to crosses the grid to the home cage on three consecutive days prior to stroke. The total number of steps and the number of foot faults on the grid was assessed. A foot fault was defined as penetration of the paw through a grid square. The performance was expressed as the mean number of faults of the right paws as the fraction of the total number steps from three tests.

Grip force test

This test informs on neuromuscular functions of the paw The rat was hand-held and allowed to voluntarily grip a T-bar (Grip Strength Test Meter GS3; Bioseb, Chaville, France). The rat was then pulled backwards and the maximal strength out of three trials recorded. This procedure was repeated three times. The force in percent of pre-stroke values was calculated.

Postural hang reflex test

This test evaluates the response to lateral push on spontaneous activity, symmetry in limb movement, forepaw outstretching, resistance and circling behavior of rats. A 4-score grading system was used: Score 3 (no deficit); Score 2 (slight deficit) failure to extend the right contralateral forelimb (cFL) when suspended by the tail; Score 1 (moderate deficit) flexion of cFL when suspended by tail and asymmetry in resistance to lateral push of the rat's body to both directions when it was on the table; Score 0 (severe deficit) twists its thorax when it was on the table (flexion of the forearm to the right part of the body), turned/circled to the contralateral (right, paretic) side or fell down, together with flexion or clenching of cFL and asymmetry in resistance to lateral pushes. A laterality index (LI) is calculated ipsilateral - contralateral push score/scor ipsilateral + contralateral score push.

Assessment of lesion size and immunohistochemistry

In brief, free-floating sections were collected throughout the anterior-posterior length of the brain for every 0.5 mm in mice and every 1 mm in rats. Sections washed and quenched in 3%

H_2O_2 and 10% MeOH for 12 min, and incubated in blocking solution for 1 hour (5% normal donkey serum and 0.25% Triton X-100), were incubated overnight at 4°C in rabbit monoclonal anti-NeuN antibody 1:5000 (MABN140, Millipore) followed by incubation in a secondary biotinylated donkey anti-rabbit antibody 1:400 (Jackson ImmunoResearch) for 1.5 hours. Visualization was achieved via a Vectorstain ABC kit (Vectorlab) and 3,3-diaminobenzidine/H_2O_2 (DabSafe, Saveen Werner). The sections were mounted and imaged using a flatbed scanner. The area of NeuN-stained tissue was determined for both hemispheres using the ImageJ software. For each section the intact tissue's area of the ipsilesional hemisphere was divided by a factor compensating for either tissue shrinkage or edema (total area of contralesional hemisphere/total area of ipsilesional hemisphere) and subtracted from the area contralesional hemisphere. Finally lesion volumes were calculated by integration. Assessment of infarct size was also evaluated paraffin sections. Brains were fixed in Carnoy's solution, embedded in paraffin. Sections of 10 µm were made regularly spaced every 550 µm throughout the extension of the ischemic region, deparaffinized and processed for staining with thionin (Nissl staining for histologic assessment of neuronal degeneration). The position of the cannulae/injection sites was assessed visually using an common rat brain atlas. The following primary antibodies were used for immunohistochemistry: Rabbit anti-Parvalbumin (PV27, Swant, Marly, Switzerland, 1:2500), Mouse anti-chondroitin sulphate proteoglycan (CAT-315, MAB1581, Millipore, 1:1000), and Mouse anti-GAD67 (MAB 5406 , clone 1G10.2, Millipore, 1:750)

Western blot analysis

Mice were anesthetized in isoflurane and decapitated. The brain was removed from the scull and immediately frozen in isopentane (Sigma-Aldrich, Taufkirchen, Germany) on dry ice. Tissue was dissected in a glove box at -20 ˚C and thereafter stored at -80 ˚C. Proteins were extracted from the frozen brain tissue and lysates were diluted in Tricine sodium dodecyl sulfate (SDS) sample buffer. Proteins were denatured at 95 °C for five minutes and 25 ug of protein were separated on 10-20% gradient Tricine gels (Novex, Invitrogen). After transferring proteins onto low fluorescence polyvinylidene difluoride membranes (Millipore), these were blocked with 5% non-fat dry milk solution in tris buffered saline with Tween 20 1% (TBS-T). The membarnes were incubated in primary antibody in 5% BSA solution in TBS-T, at 4 °C overnight. The following primary antibodies were used: Rabbit anti-GluR5 (#AB5675, Millipore, 1:5000), Mouse anti-GAD67 (MAB 5406, clone 1G10.2, Millipore, 1:4000); Rabbit polyclonal anti-GAD65 (ABN101, Sigma-Aldrich, 1:4000); Rabbit anti-Parvalbumin (PV27, Swant, Marly, Switzerland, 1:2000). Blots were subsequently incubated with StarbrightBlue 700 fluorescent secondary anti-rabbit (Bio-Rad, cat#12004162, 1:5000) and with Rhodamine anti-GAPDH

antibody (Bio-Rad, cat#12004168, 1:10000), for one hour at room temperature in the blocking solution. Signals were visualized by using a Chemidoc MP system (Bio-Rad, Solna, Sweden). Densiometric measurements of the protein of interest were normalized to GAPDH expression using Image Lab 5.0 software (Bio-Rad) and presented as arbitrary units.

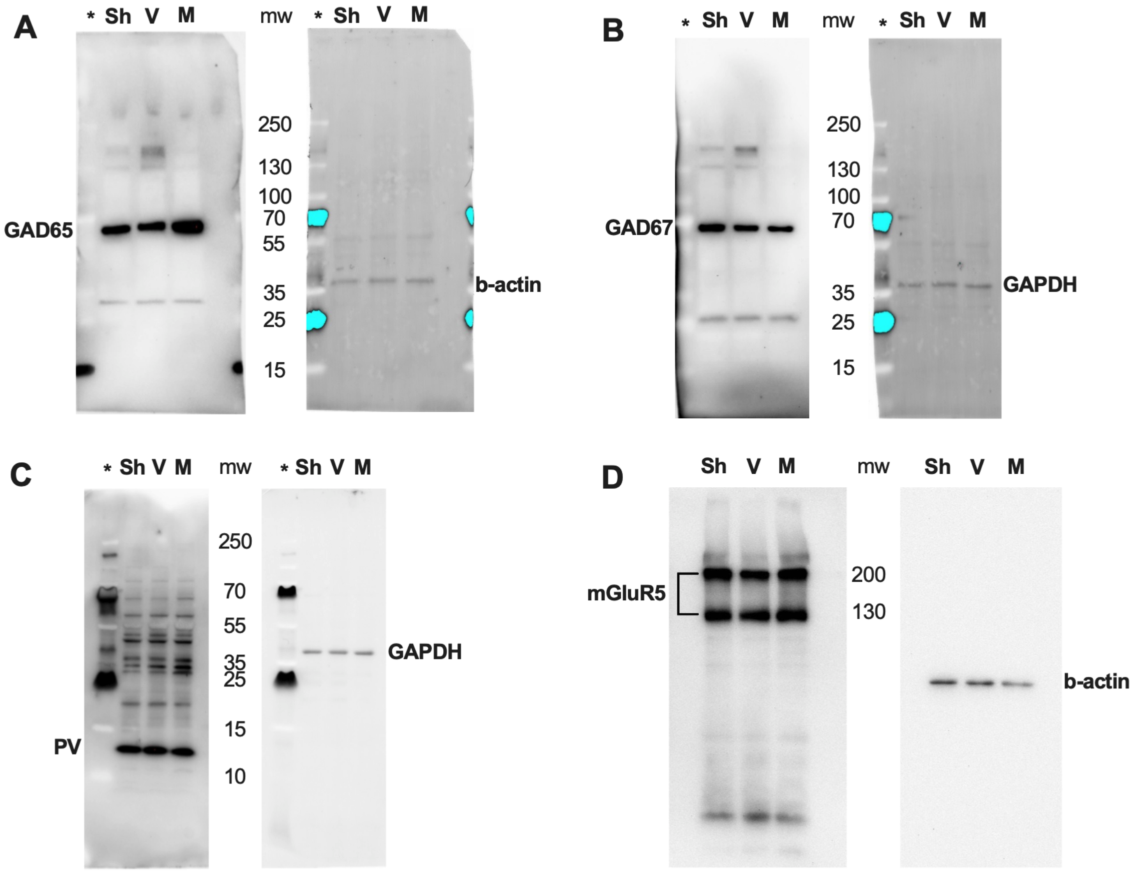

Supplementary Figure 6 Western blots of full-length gels. (A) GAD65, (B) GAD67, (C) parvalbumin (PV) and (D) mGluR5 in tissue homogenates of contralateral somatosensory cortex after PT-stroke and 14 days of recovery. Lanes are indicated as follows: molecular markers (*), Sham-operated (Sh), Vehicle-treated (V) and MTEP-treated (M) mice. Molecular weights (mw) are indicated.

Statistical analysis

Experimental data are generally classified into parametric or non-parametric categories, and the statistical methods employed are summarized in Supplementary Table 4. Group differences in infarct sizes and other unpaired, parametric data sets (adhesive removal test, grip test, beam walk test) were evaluated by 2-tailed t-test or one-way analysis of variance (ANOVA) followed by post hoc, 2 tailed t-tests, corrected for multiple comparisons using Sidak's correction. Group differences in the grid test were assessed using paired, 2-tailed t-tests. Group differences in non-parametric paw placement scores were evaluated using Mann

Whitney's U-test for comparing 2 groups, or the Kruskal-Wallis test if more than 2 groups were compared followed by Dunn's post hoc test. Wilcoxon's matched-paired signed test was used to evaluate performance on the postural hang rung laterality test.

For all RSFC data, Pearson-R correlation coefficients were converted to Fisher-Z values prior to group averaging or statistical testing. Group differences in clustering coefficient and path length were assessed via one-way ANOVA followed by false discovery rate (FDR) correction. Statistical comparisons of node degree between groups in multiple regions were performed using a two-way ANOVA with treatment group serving as one variable and brain region as a repeated measure, and followed by FDR correction. The high dimensionality of some RSFC datasets (Fig. 5) results in a multiple comparisons problem (~100 million comparisons) and required a non-parametric means for assessing statistical significance of the PCs. Extensive permutation resampling (3000 iterations) of all mice between groups was used to determine the amount of variance expected in the first eigenvalue following PCA decomposition in the null case. In the true eigenspectrum (i.e. FC differences between MTEP PT and Vehicle PT mice), the variance explained by PC1 was in the 97.45th percentile (i.e., $p=0.0255$) and was considered statistically significant.

Sample size, effect size and power calculations

Sample sizes were based on power calculations (G*Power, University of Dusseldorf, (1)) using effect sizes from pilot and previous experiments.

<u>Resting state functional connectivity.</u> Group sizes for neuroimaging studies were calculated based on effect sizes and group variance from our previous experiments (2). For interhemispheric connectivity (M1 region) standard deviations ranged between 20%-24% of the mean across groups; difference in mean 38%. For intrahemispheric node-degree (right M2 region), the difference in the mean was 30% and standard deviations ranged between 19,5-23,5% of mean across groups; α: 0.05%; power ($1-\beta$): 80%). A minimum group size of $n=10$ was derived for both experiments.

<u>Behavior.</u> In the rat, exploratory experiments indicated that treatment with MTEP provided a strong recovery enhancing response in the PP test of similar magnitude as seen earlier in our EE studies (3,4). For a first approximation of the sample size in the rat MTEP experiments we therefore used pooled PP data from unpublished, previous published (3, 4) studies on the effect of EE at 14 days after stroke, Figure 7A.

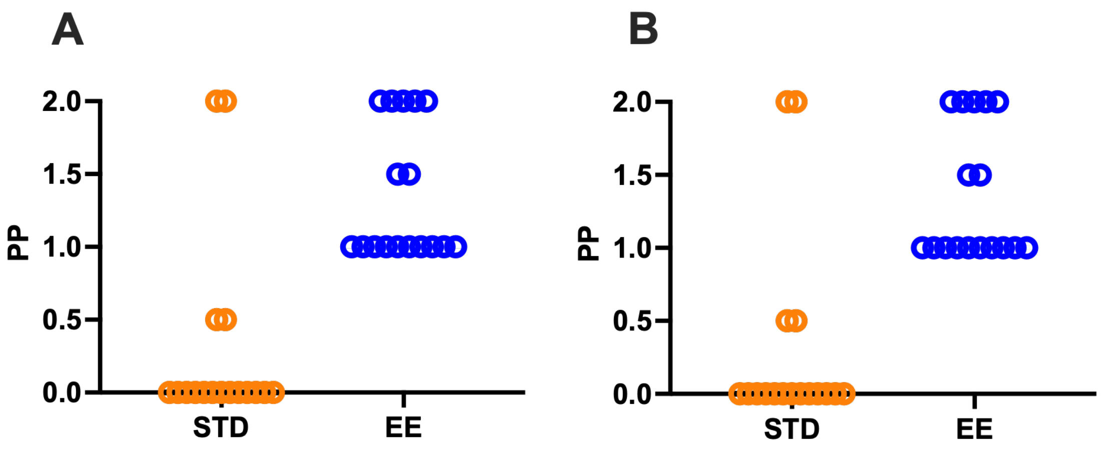

Supplementary Figure 7. The effect of enriched environment on paw placement after stroke. (A) Pooled paw placement data on the effect of enriched environment (EE, n=17) compared to standard environment (STD, n=19) at 14 days after PT stroke in rats. **(B)** Pooled paw placement data on the effect of enriched environment (EE, n=25) compared to standard (STD, n=27) at 7 days after PT stroke in mice.

Using the G*Power calculator, and data reported in Supplemental Figure 7A an effect size of 1,86 was derived. With this effect size and a power of 80% and p=0,05, a sample size of 6 was calculated, which guided us in sample size selection in the MTEP rat experiments. Using the data reported in Fig 1D of the present manuscript, we obtained an effect size of 2,57 and power of 99%, when comparing the vehicle and MTEP treated groups at 14 days post. For a power of 80% a minimum sample size of 4 was calculated. From the data in Fig. 1F (n=4 in each group) of the present manuscript, we calculated an effect size of 3,68 and power of 90%.

In analogy with the rat experiments we assessed the effect size of EE in mice experiments based on earlier data (2, 5) and pilot experiments, Supplementary Figure 7B. An effect size of 1,76 and a power of 99% was obtained, and for a power of 80% a sample size of 6 was derived that guided us in the selection of sample size in the first mouse MTEP series (Figure 3C). In this experiment an effect size of 5,21 and a power of 99% was obtained. Using this effect size, a minimum sample size of 3 is required to detect group wise differences with a power of 80%, which guided us in sample size selection in subsequent studies.

References

1. Prajapati B, Dunne M, Armstrong R. Sample size estimation and statistical power analyses. *Optom Today*. 2010;16:10–8.

2. Hakon J, Quattromani MJ, Sjolund C, et al. Multisensory stimulation improves functional recovery and resting-state functional connectivity in the mouse brain after stroke. *Neuroimage Clin*. 2018;17:717-730.

3. Madinier A, Quattromani MJ, Sjolund C, Ruscher K, Wieloch T. Enriched housing enhances recovery of limb placement ability and reduces aggrecan-containing perineuronal nets in the rat somatosensory cortex after experimental stroke. *PLoS One*. 2014;9:e93121.

4. Quattromani MJ, Pruvost M, Guerreio C, Backlund F, Englund E, Aspberg A, Jaworski T, Christensen JH, Ruscher K, Kaczmarek L, Vivien D, Wieloch T (2017) Extracellular matrix modulation is driven by experience-dependent plasticity during stroke recovery. Molecular Neurobiology. Mar 13. doi: 10.1007/s12035-017-0461-2.

5. Quattromani MJ, Cordeau P, Ruscher K, Kriz J, Wieloch T. Enriched housing down-regulates the Toll-like receptor 2 response in the mouse brain after experimental stroke. *Neurobiol Dis*. 2014;66:66-73.
